# Estimating the Integrated Information Measure Phi from High-Density Electroencephalography during States of Consciousness in Humans

**DOI:** 10.3389/fnhum.2018.00042

**Published:** 2018-02-16

**Authors:** Hyoungkyu Kim, Anthony G. Hudetz, Joseph Lee, George A. Mashour, UnCheol Lee, the ReCCognition Study Group, Michael S. Avidan

**Affiliations:** ^1^Department of Anesthesiology, University of Michigan Medical School, Ann Arbor, MI, United States; ^2^Center for Consciousness Science, University of Michigan Medical School, Ann Arbor, MI, United States; ^3^Neuroscience Graduate Program, University of Michigan, Ann Arbor, MI, United States

**Keywords:** integrated information theory, Φ, functional connectivity, electroencephalography, consciousness, anesthesia, human

## Abstract

The integrated information theory (IIT) proposes a quantitative measure, denoted as Φ, of the amount of integrated information in a physical system, which is postulated to have an identity relationship with consciousness. IIT predicts that the value of Φ estimated from brain activities represents the level of consciousness across phylogeny and functional states. Practical limitations, such as the explosive computational demands required to estimate Φ for real systems, have hindered its application to the brain and raised questions about the utility of IIT in general. To achieve practical relevance for studying the human brain, it will be beneficial to establish the reliable estimation of Φ from multichannel electroencephalogram (EEG) and define the relationship of Φ to EEG properties conventionally used to define states of consciousness. In this study, we introduce a practical method to estimate Φ from high-density (128-channel) EEG and determine the contribution of each channel to Φ. We examine the correlation of power, frequency, functional connectivity, and modularity of EEG with regional Φ in various states of consciousness as modulated by diverse anesthetics. We find that our approximation of Φ alone is insufficient to discriminate certain states of anesthesia. However, a multi-dimensional parameter space extended by four parameters related to Φ and EEG connectivity is able to differentiate all states of consciousness. The association of Φ with EEG connectivity during clinically defined anesthetic states represents a new practical approach to the application of IIT, which may be used to characterize various physiological (sleep), pharmacological (anesthesia), and pathological (coma) states of consciousness in the human brain.

## Introduction

Integrated information theory (IIT) postulates that consciousness is identical to integrated information and that a system's capacity for consciousness can be expressed by a quantitative measure referred to as Φ (Tononi, 2004; Oizumi et al., 2014, 2016a,b; Tononi et al., 2016). Specifically, it has been postulated that the loss and recovery of consciousness are associated with, respectively, the breakdown and restoration of integrated information in the brain (Alkire et al., 2008; Lee et al., 2009; Tononi, 2010; Tononi and Koch, 2015). This prediction should hold true for physiological (slow-wave sleep), pharmacological (anesthesia), and pathological (coma) states of unconsciousness. Thus, far, only surrogates of integrated information have been amenable to quantitative analysis due to the explosive computational demand associated with calculating Φ for real systems of interest including the brain. For instance, to calculate Φ from a 128-channel electroencephalogram (EEG), we would need to find the bipartition of 128-channels that has the maximally irreducible integrated information among all possible bipartitions, that is, $\sum_{k=1}^{64}\;C\left(128,k\right)≅1.8^{*}10^{38}$ bipartitions of the EEG channels (the binomial coefficient *C* (128, *k*) denotes the number of choices of *k* EEG channels from 128 channels). Several attempts have been made to overcome the computational limitations (Tegmark, 2016; Krohn and Ostwald, 2017; Toker and Sommer, 2017). However, they have not provided sufficient evidence for IIT because they did not compare the actual change of Φ during different levels of human consciousness.

**Figure 1:**
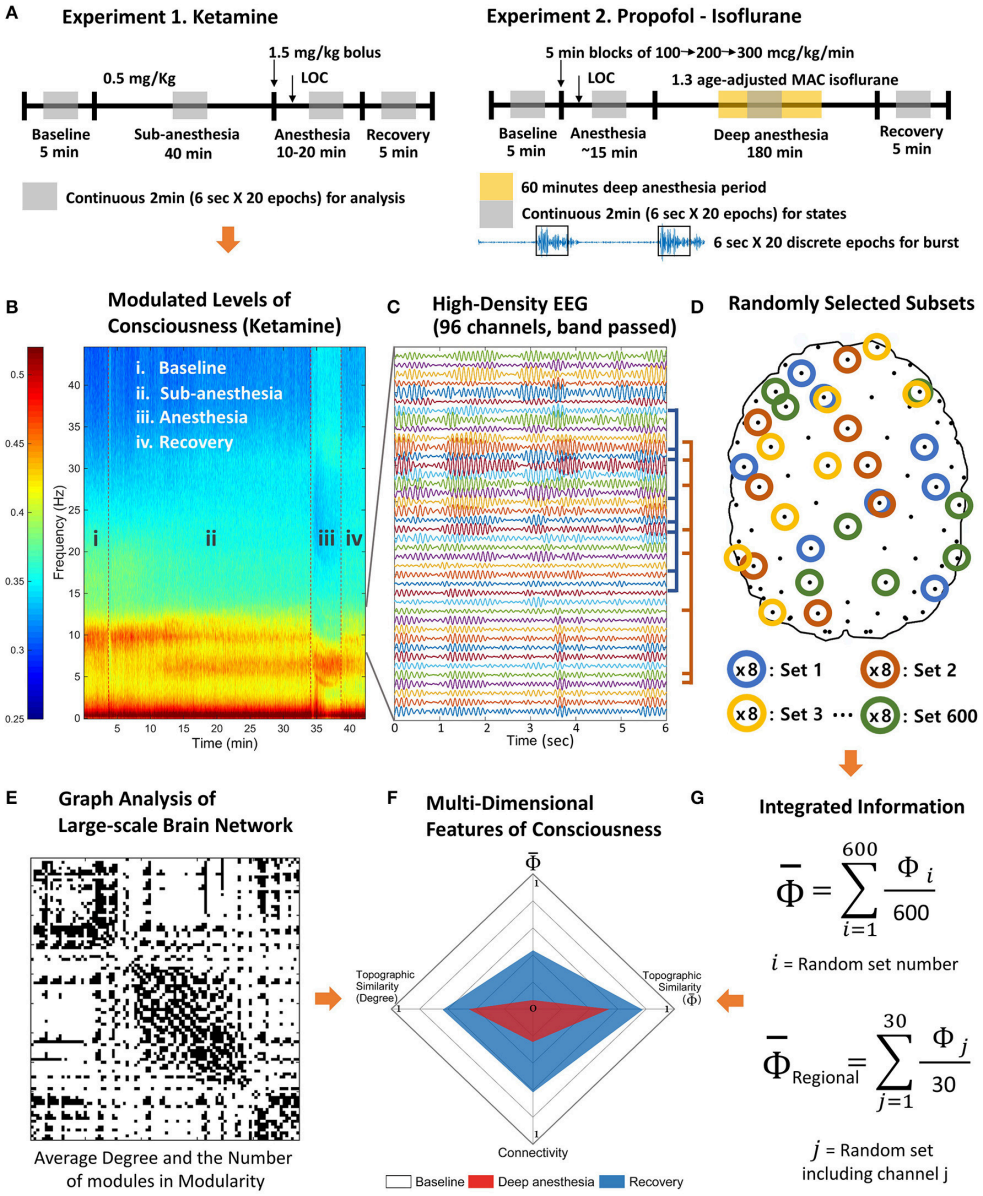
Schematic diagram of experiments and analysis. The high-density EEG data were recorded from two experiments. **(A)** Experiment 1 includes baseline, subanesthetic doses of ketamine, ketamine anesthesia, and recovery. Experiment 2 includes baseline, induction of general anesthesia with propofol, deep anesthesia (burst suppression) with isoflurane (1.3 age-adjusted minimum alveolar concentration [MAC]), and recovery. **(B)** Based on the power spectrum, 2 min continuous signals were selected in the middle of each state of consciousness. **(C)** The selected periods were divided into 20 epochs of 6 s and band pass filtered to delta, theta, alpha, beta, and gamma frequency range. **(D)** After preprocessing, 600 sets of eight EEG channels were randomly selected to estimate the average Φ of each EEG channel and contribution of each EEG channel on the average Φ (regional Φ). **(E)** The degree and the number of modules of the functional connectivity network were measured for each state. **(F)** The multi-dimensional parameter space was constructed with the degree of functional connectivity, the topographic similarity of node degrees, average Φ, and the topographic similarity of regional Φ estimated with random selections in **(G)**.

In this study, we introduce a novel method to estimate the relative change of Φ and test its ability to predict levels of consciousness as modulated by various anesthetic agents (see Figure 1 for schematic diagram of experiment and analysis; see Table 1 for terminology). Considering both the computational limitation of Φ for a whole system and the fact that EEG reflects superficial brain activity, the method was devised to investigate the relative change of the mean integrated information across states and its relationship with conventional EEG properties, rather than calculating the precise Φ of a system. The mean integrated information (denoted as $\bar{\Phi }$) was estimated from many small sets of EEG channels randomly and globally sampled from high-density EEG. This method allowed us to determine the contribution of each EEG channel to the estimated $\bar{\Phi }$ and derive a topographic structure of regional Φ. We compared the effects of three different anesthetics (ketamine, propofol, and isoflurane) with distinct pharmacological and neurophysiological profiles in five states (conscious, sedated, non-responsive, non-responsive with burst suppression, and recovery) on the global $\bar{\Phi }$, topographic structure of regional Φ, and conventional EEG band powers that are frequently used empirically to index the level of consciousness. We also examined the relationship of $\bar{\Phi }$ to EEG network properties such as the strength and topology of functional connectivity. Finally, the five states of consciousness were represented as shapes in a multi-dimensional parameter space that consisted of four parameters related to $\bar{\Phi }$ and EEG connectivity. We found that, in contrast to the component variables, only the multi-dimensional parameter space was able to properly differentiate all anesthetic states.

**Table 1:** Glossary.

**Terms**	**Definition**
Integrated information (Φ)	Information that is specified by a system that is irreducible to the information specified by its parts. It is calculated as the distance between the conceptual structures specified by the intact system and that specified by its minimum information partition.
Effective information (φ)	The repertoire specified by a mechanism in a state informs the possible past and future states of a system. Effective information is defined as the distance between effect repertoire and corresponding unconstrained probability distributions.
Markovian integrated information (Φ_*DM*_)	Integrated information for discrete dynamic systems. Each partitioned state is measured with the reduced entropy by maximizing the entropy of the past state.
Empirical integrated information (${\tilde{\Phi }}_{E}$)	Integrated information for a continuous time series derived from Markovian integrated information by using the assumption of a Gaussian distribution of time series and differential entropy formula.
Auto-regressive integrated information (${\tilde{\Phi }}_{AR}$)	Integrated information for systems with a non-Gaussian distribution of time series by substituting the covariance of the time series of the empirical integrated information to the prediction error of linear regression of time series.
Mismatched decoding integrated information (Φ^*^)	The Φ^*^ is calculated with the difference between actual mutual information under the actual probability distribution of the system and the hypothetical mutual information under mismatched probability distribution where a system is partitioned into hypothetical independent parts.
Relative changes of integrated information ($\bar{\Phi }$)	Estimation of the mean integrated information of randomly and globally sampled small sets of EEG channels. This method was introduced in the current study to investigate the relative change of the mean integrated information across states and its relationship with conventional EEG properties, rather than calculating the precise Φ of a system.
Regional integrated information (${\bar{\Phi }}_{R}$)	Evaluation of the contribution of a certain EEG channel to the $\bar{\Phi }$ of the whole EEG channels. The contribution is evaluated with the following assumption: if the sets of EEG channels that include a certain EEG channel have larger $\bar{\Phi }$, the specific EEG channel probably contributed to the $\bar{\Phi }$ of the whole EEG with a larger weight.

## Methods

### Ethics statement

This study was conducted at the University of Michigan Medical School and approved by the Institutional Board Review (HUM00061087 and HUM0071578); written informed consent was obtained from all participants.

### Experimental procedures

In this study we conducted secondary analyses of high-density EEG data gathered in healthy volunteers during two independent studies; detailed methodology can be found in Vlisides et al. (Vlisides et al., 2017) and Blain-Moraes et al. (Blain-Moraes et al., 2017; Maier et al., 2017). After the approval of the Institutional Review Board and written informed consent, 19 human subjects were studied on two separate experiments with 128-channel EEG. In 10 subjects, four states were investigated: (1) baseline consciousness, (2) subanesthetic dose (sedated state), 0.5 mg/kg ketamine administered over 40 min, (3) general anesthesia (non-responsive state), defined as loss of response to the verbal command “squeeze your left [or right] hand twice,” after a single bolus dose of 1.5 mg/kg ketamine, and (4) recovery, defined as return of responsiveness. In nine additional subjects, the following four states were investigated: (1) baseline consciousness, (2) general anesthesia (non-responsive state), defined as loss of response to a verbal command after an induction sequence of propofol (100 mcg/kg/min for 5 min, followed by 200 mcg/kg/min for 5 min, followed by 300 mcg/kg/min for 5 min), (3) deep anesthesia (non-responsive state with burst suppression), induced by 1.3 age-adjusted minimum alveolar concentration of isoflurane, and (4) recovery, defined as return of responsiveness.

EEG was acquired with 128-channel HydroCel nets, Net Amps 400 amplifiers, and Net Station 4.5 software (Electrical Geodesics, Inc., USA). The EEG was digitized continuously at 500 Hz with a vertex reference. Per manufacturer recommendations, channel impedances were kept at <50 kΩ, and the net was wrapped with gauze to optimize contact between the electrodes and scalp. Baseline and recovery EEGs in the ketamine experiments were recorded for 5 min. EEG during exposure to subanesthetic ketamine was recorded during an infusion of 0.5 mg/kg administered over 40 min. EEG during ketamine anesthesia was recorded after an intravenous bolus of 1.5 mg/kg ketamine until return of responsiveness. Baseline and recovery EEG in the propofol-isoflurane experiments was recorded for 5 min. The propofol administration sequence was recorded for 15 min. Deep anesthetic state EEG with isoflurane was recorded for ~180 min. After the recording, 32 channels on the lower part of the face and head were removed, leaving 96 channels in place, to avoid confounds in the analysis of occipital and prefrontal channels due to contamination from contact with the bed and facial movements. The average reference was used for referencing and the windowed sinc-FIR filter was used to avoid a possible shifting of the signal phases (in the MATLAB toolbox from EEGLAB). For EEG analysis, we chose a clean 2-min EEG epoch for each state (baseline, sedated, anesthetized, and recovery), and segmented it into 6-s long EEG epochs. Noisy epochs were excluded by visual inspection based on power spectrum and EEG signal.

In this study, we differentiated deep anesthesia (non-responsive with burst suppression) from general anesthesia (non-responsive state). Burst suppression is a well-known EEG characteristic observed in the deeply anesthetized state. The EEG pattern is characterized by periods of high voltage electrical activities alternating with minimal activity. We used the burst suppression ratio (BSR: the fraction of EEG in suppression per epoch) of 0.3 to determine the burst suppression period (Vijn and Sneyd, 1998). We chose 20 burst suppression epochs, each of which was long enough for analysis (>10 s), from the subjects who showed burst suppression (5 of 9 subjects during isoflurane). Each burst suppression period was separated again into burst and suppression periods. Considering the transient state and to match the data length with the other states, we chose the first 6 s of both periods for the analysis.

### Spectrogram analysis

For all selected periods within each subject, spectral power was computed based on the short-time Fourier transform using the “spectrogram.m” function in the MATLAB Signal Processing Toolbox (time window: 3 s hamming window, overlap: 50%). The relative power was then computed for each experimental period at each of five frequency bands (delta: 0.1–4 Hz, theta: 4–8 Hz, alpha: 8–13 Hz, beta: 13–25 Hz, gamma: 25–45 Hz), for all 96 channels. The difference in relative power among the different states was tested with linear mixed model analysis.

### Calculation of Φ

The integrated information theory defines *integrated information* (Φ) as the effective information (φ) of the minimum information partition (MIP) in a system (Tononi, 2004, 2012; Oizumi et al., 2014, 2016a; Tononi et al., 2016). The MIP is also defined as the partition having minimum effective information among all possible partitions.

(1) Φ[X;x]=:φ[X;x, MIP(x)]

(2) MIP (x)=:arg min{φ(X;x, P)}

where X is the system, x is a state, and P is a partition P = {*M*^1^, …, *M*^*r*^}.

Identifying the MIP requires searching all possible partitions and comparing their effective information φ. This is the most time consuming process in the application to high density EEG. Furthermore, considering the fact that the EEG recorded during anesthetic state transitions are non-Gaussian and continuous time series, it is important to choose a valid version of Φ (Φ_*DM*_, ${\tilde{\Phi }}_{E}$, ${\tilde{\Phi }}_{AR}$, Φ^*^). In Table 1, we summarized all versions of Φ we considered in this study as well as the new approach described.

Markovian integrated information (Φ_*DM*_) is defined for a discrete dynamic system (Balduzzi and Tononi, 2008). The effective information (φ_*DM*_) is measured with the reduced entropy by maximizing the entropy of the past state; the lowest value of effective information among the partitions is defined as Φ_*DM*_.

(3) $$\varphi_{DM}[X;\;\{M^{1},\;\ldots ,\;\;M^{r}\;\}]\equiv {\sum^{\;}}_{k=1}^{r}H(M_{0}^{k}|M_{1}^{k})-H(X_{0}|X_{1})$$

where X is a discrete system, and M represents the subsets. Barrett and Seth modified Φ_*DM*_, and introduced empirical integrated information ($\tilde{\Phi }$_*E*_), which works for a continuous time series. $\tilde{\varphi }$ is the integrated information for a bi-partition;

(4) $$\tilde{\varphi }[X;\tau ,\{M^{1},M^{2}\}]=:{\sum^{\;}}_{k=1}^{2}H\;\left(M_{t-\tau }^{k}|M_{t}^{k}\right)-H(X_{t-\tau }|X_{t}),$$

where τ is the time delay between the past and present states, and *M*^1, 2^ are the bi-partitioned subsets. To make the calculation of (4) easier, the entropy terms *H* are substituted with the covariance term ∑ with the differential entropy formula (5). ${\tilde{\varphi }}_{E}$ is the empirical integrated information for a bi-partition (Barrett and Seth, 2011), which works for Gaussian time series.

(5) $$\begin{array}{l} \begin{array}{c} H(X_{t-\tau }|X_{t})=\frac{1}{2}\log[det\sum^{\;}(X_{t-\tau }|X_{t})] \end{array}\\ \;\begin{array}{c} +\;\frac{1}{2}\;nlog\;(2\pi \;2\pi e) \end{array} \end{array}$$

(6) $$\begin{array}{l} {\tilde{\varphi }}_{E}[X;\tau ,\{M^{1},M^{2}\}]=:{\sum^{\;}}_{k=1}^{2}\frac{1}{2}\log\left\{det\sum^{\;}(M_{t-\tau }^{k}|M_{t}^{k})\right\}\\ \;\begin{array}{c} -\;\frac{1}{2}\log\left\{\det\sum^{\;}(X_{t-\tau }|X_{t})\right\} \end{array} \end{array}$$

For the application to non-Gaussian time series, ${\tilde{\varphi }}_{E}$ is reformulated with auto-regression of the time series, substituting the covariance ∑ in Equation (6) with the prediction error of linear regression. ${\tilde{\varphi }}_{AR}$ for a bi-partition is defined as following;

(7) $$\begin{array}{l} {\tilde{\varphi }}_{AR}[X;\tau ,\{M^{1},M^{2}\}]=:{\sum^{\;}}_{k=1}^{2}\frac{1}{2}\log\left\{det\sum^{\;}(E^{M^{k}})\right\}\\ \begin{array}{c} \;-\;\frac{1}{2}\log\{\det\sum^{\;}(E^{X})\}, \end{array} \end{array}$$

where τ is the time delay between the past and present state*s*, and *M*^1, 2^ are the bi-partitioned subsets. *E* is the prediction error of linear regression, *X*_*t*_ = α + A·*X*_*t*−τ_ +*E*_*t*_. *det*∑(*E*^*M*^*k*^^) is the determinant of the covariance (∑) of predictions errors (*E*^*M*^*k*^^). The ${\tilde{\varphi }}_{AR}$ of MIP for a given system is defined as ${\tilde{\Phi }}_{AR}$, and, notably, ${\tilde{\Phi }}_{AR}$ can be applied to a non-Gaussian and continuous time series. Thus, in this study, we used ${\tilde{\Phi }}_{AR}$ developed by (Barrett and Seth, 2011), which is applicable to both Gaussian or non-Gaussian EEG data recorded under various states of consciousness. However, ${\tilde{\Phi }}_{AR}$ does not solve the problem of computation time. In the next section, we describe a method to estimate the relative change of ${\tilde{\Phi }}_{AR}$ across states based on high-density EEG. From here, we denote ${\tilde{\Phi }}_{AR}$ of EEG as Φ for convenience.

### Estimation of the relative change of Φ across various states of consciousness

Although many improvements have been made in the algorithms of Φ over the last decade, the computation time is still unrealistic because of the need to search an enormous number of partitions to identify the MIP.

Here we suggest a method to circumvent the fundamental problem by considering the average features of many small sample units rather than trying to identify the MIP and its effective information for all EEG channels. A sample unit consists of a small number of EEG channels randomly selected with the total number of sample units large enough to represent the behavior of the entire high-density EEG montage. In this study, we directed our interest only to the relative changes of Φ values across states (baseline, sedation, anesthesia, burst, suppression, and recovery), rather than to the exact Φ value of the brain for each state, which is impossible with the superficial and spatially imprecise brain activity recorded by EEG.

For each sample unit, we were able to calculate the MIP and its effective information φ, that is, the Φ of the sample unit. For instance, in this study, we selected 8 random EEG channels for a sample unit, and acquired 600 sample units that were randomly selected from the baseline states. The same 600 sample units determined in the baseline were then compared across states to investigate the increase or decrease of Φ values. Since the number of possible bipartitions of 8 channels is $\sum_{k=1}^{4}\;C\left(8,k\right)=162$, where *C* stands for the combination of *k* unique elements chosen from 8 possible elements (Figure 1D), the Φ calculations for various frequency bands and states of all subjects in two anesthetic experiments are possible in a relatively short computational time period.

The average $\bar{\Phi }$ is defined as follows.

(8) $$\bar{\Phi }=\frac{1}{k}{\sum^{\;}}_{i=1}^{k}\Phi_{i}(\text{n})-\frac{1}{k}{\sum^{\;}}_{i=1}^{k}\;median(\Phi_{surr(i)}(\text{n}))$$

where *n* is the number of EEG channels for a sample unit and *k* is the number of sample units of the *n* EEG channels. Φ_*i*_(*n*) measures the effective information of MIP for the *n* EEG channels, by definition, the integrated information of the sample unit. Here, we chose *n* = 8 and *k* = 600 for the given data, and tests for validity are described in the next section. Φ_*surr*_(*n*) is the spurious Φ_*i*_(*n*) estimated from randomly shuffled 20 EEG data sets. Subtracting the randomness, $\bar{\Phi }$ reflects the average integrated information of 600 sample units taken from the high-density EEG data that surpasses the spurious integrated information from the surrogate data.

### Reliability test for the random EEG channel selection

Since the random selection of EEG channels can produce variable $\bar{\Phi }$, we need to determine the appropriate number of sample units that reliably represent a given EEG data. Here, we measured the coefficient of variance, the ratio of variance against the mean value, for different numbers of sample units (McLachlan, 1978; Faber and Korn, 1991). We fixed the number of EEG channels as 8 for a sample unit and changed the total number of sample units from 10 to 600. The calculation of $\bar{\Phi }$ for a certain number of sample units was repeated 300 times to evaluate the coefficient of variance with the mean and variance.

(9) $${\bar{\Phi }}_{k}=\{{\bar{\Phi }}_{k,1},{\bar{\Phi }}_{k,2},{\bar{\Phi }}_{k,3},\ldots ,\;{\bar{\Phi }}_{k,300}\})$$

(10) $$\text{Coefficient of Variance}\;({\bar{\Phi }}_{k})=\frac{\sigma \;({\bar{\Phi }}_{k})}{<{\bar{\Phi }}_{k}>},$$

where the total number of the sample *k* is changed from 10 to 600. The coefficient of variance is decreased with an increase in the total number of sample units. For *k* = 600 the coefficient is smaller than 0.01 (Supplementary Figure 1). This result indicates that when we use the 600 sample units and 8 random EEG channels for each sample unit to estimate $\bar{\Phi }$, the variance of the estimated $\bar{\Phi }$ over 300 iterations is <1% of the mean. In other words, the estimation of $\bar{\Phi }$ with the total 600 sample units and 8 random EEG channels for a sample unit gives us a stable value close to the mean, which can be deemed to represent reliably the increase or decrease of $\bar{\Phi }$ for high-density EEG data across states.

### Estimation of regional ${\bar{\Phi }}_{r}$

We also estimated the contribution of a single EEG channel *i* on $\bar{\Phi }$ based on the assumption that if many sample units including a specific channel *i* have larger Φ values, then the channel *i* probably contributes to the ${\bar{\Phi }}_{\;}$ with a larger weight than the other channels. Here, the contribution of an EEG channel *i* to the $\bar{\Phi }$, denoted as ${\bar{\Phi }}_{R\left(i\right)}$, should be differentiated from the estimation of $\bar{\Phi }$ for 96 channel EEG. With the ${\bar{\Phi }}_{R\left(i\right)}$ of each EEG channel, we constructed the topography of ${\bar{\Phi }}_{R}$, which enabled us to examine the degree to which a given region contributes to $\bar{\Phi }$. To find the appropriate number of sample units for a stable ${\bar{\Phi }}_{R\left(i\right)}$, we evaluated the coefficients of variance for different number of sample units *k* with 8 EEG channels per a sample unit. With *k* = 30, the coefficient of variance becomes <0.05 (Supplementary Figure 2). In this study, we determined the number of sample units to estimate reliable $\bar{\Phi }$ and ${\bar{\Phi }}_{R}$, that is, 600 sample units of 8 EEG channels for $\bar{\Phi }$ and 30 sample units of 8 EEG channels for ${\bar{\Phi }}_{R\left(i\right)}$. The number of EEG channels as 8 for a sample unit was determined by considering the robustness and computational time together for the given data.

### Graph theoretic network analysis

To reconstruct a network from EEG data, we used weighted Phase Lag Index (wPLI), which is robust to the EEG volume conduction problem (Stam et al., 2007; Vinck et al., 2011). The wPLI is a measure of phase locking between two EEG signals, which improves Phase Lag Index (PLI) with weights of the imaginary part of cross-spectrum, reducing noise and volume conduction effects. When the imaginary part of cross-spectrogram is ℑ[*X*],

(11) $$\text{wPLI}\;\equiv \frac{\left|E\{ℑ\{X\}\}\right|}{E\{|ℑ\{X\}|\}}=\frac{\left|E\{|ℑ\{X\}|sgn(ℑ\{X\})\}\right|}{E\{|ℑ\{X\}|\}},$$

where the signed PLI is the numerator normalized by denominator, the imaginary part of cross-spectrum.

To reduce spurious connectivity of EEG, 20 surrogate data sets were generated with a random shuffling method, in which a time point is randomly chosen in each EEG channel; the EEG epochs are then shuffled before and after the time point. The shuffled data has almost the same amplitude distribution and power spectrum of the original EEG but disruptions of the original connectivity between two EEG signals. The non-zero wPLI from the shuffled data is regarded as spurious connectivity. We expected that different EEG frequency bands and different states would have different levels of spurious connectivity (Lee et al., 2012). Thus, after subtracting the median wPLI of 20 surrogate data sets, if the remaining wPLI was larger than 0.35, the connectivity of two EEG signals was set as 1, otherwise, it was set as 0. The threshold (0.35) was chosen to avoid an isolated node in the EEG network in the baseline states. The basic EEG network properties were examined across states during the two anesthetic experiments. The node degree of an EEG channel was defined by the numbers of links in the network. The high degree nodes in a network were deemed as hubs with the expectation that they play a more important role for integrating information (Boccaletti et al., 2006; van den Heuvel and Sporns, 2013). We examined the degree distributions in the EEG networks across states, and compared them with the regional contribution ${\bar{\Phi }}_{R}$, with the expectation that the hubs integrate more information in the brain network. We also investigated how anesthetics functionally segregated the normal EEG networks, which results in a deviation of the network from a presumed optimal balance between functional segregation and integration in the conscious state. Modularity measures the functional segregation of an EEG network and is defined by the concentration of the number of links within modules compared to that of a random network (Newman, 2006).

(12) $$\begin{array}{l} \text{Modularity }(Q)=\frac{1}{4m}\sum_{ij}\left(A_{ij}-\frac{k_{i}k_{j}}{2m}\right)\left(s_{i}s_{j}+1\right)\\ \;=\frac{1}{4m}\sum_{ij}\left(A_{ij}-\frac{k_{i}k_{j}}{2m}\right)s_{i}s_{j} \end{array}$$

where *k*_*i*_ and *k*_*j*_ are the node degrees, m is the total number of links in the network, and *s*_*i*_ = 1 if node *i* belongs to group 1 and *s*_*i*_ = −1 if it belongs to group 2 for a particular division of the network into two groups.

### Construction of multi-dimensional parameter space

The overall strengths of ${\bar{\Phi }}_{R}$ and EEG connectivity were normalized by the maximum and minimum values of the baseline (0–1 in y-axis of **Figure 5**), and the topographic similarities of ${\bar{\Phi }}_{R}$ and EEG connectivity of a state were normalized from −1 to 1 (x-axis of **Figure 5**). For the normalization, we used the baseline state as the reference state assuming that the baseline has the largest $\bar{\Phi }$ and an optimal structure of EEG connectivity for information transfer compared to the anesthetized and recovery states. Thus, the maximum values (1 s) in the x- and y-axes correspond to the baseline. The topographic similarity was evaluated with the Pearson correlation coefficient between the channel values of baseline and other states.

### Statistical analysis

Linear mixed-effects model analysis was used to evaluate the significance of the results (Pinheiro and Bates, 2000; Baayen et al., 2008). The standard form of a linear mixed-effects model is

(13) $$\begin{array}{c} Y\;=\;\text{X}\beta \;+\;\text{Zu}\;+\;\varepsilon \end{array}$$

where Y is the known vector of observation, β is an unknown vector of fixed effects, u is the unknown vector of random effects, ε is an unknown vector of random errors, and X and Z are known design matrices relating the observations Y to β and u, respectively. The analyzed properties (integrated information, degree, number of modules, and relative power) were set as observation vector Y, the states and epochs were set as fixed effect, and the epochs with subject numbers were set as random effects. The MATLAB function ‘fitlme.m’ was used to test all states pair-wise. Data from experiment 1 (ketamine) and experiment 2 (propofol-isoflurane) were calculated separately. Performance of the statistical tests was assisted by Consulting for Statistics, Computing & Analytics Research (CSCAR, University of Michigan).

## Results

### Configurations of $\bar{\Phi }$ over five EEG frequency bands for various states of human consciousness

**Figure 2:**
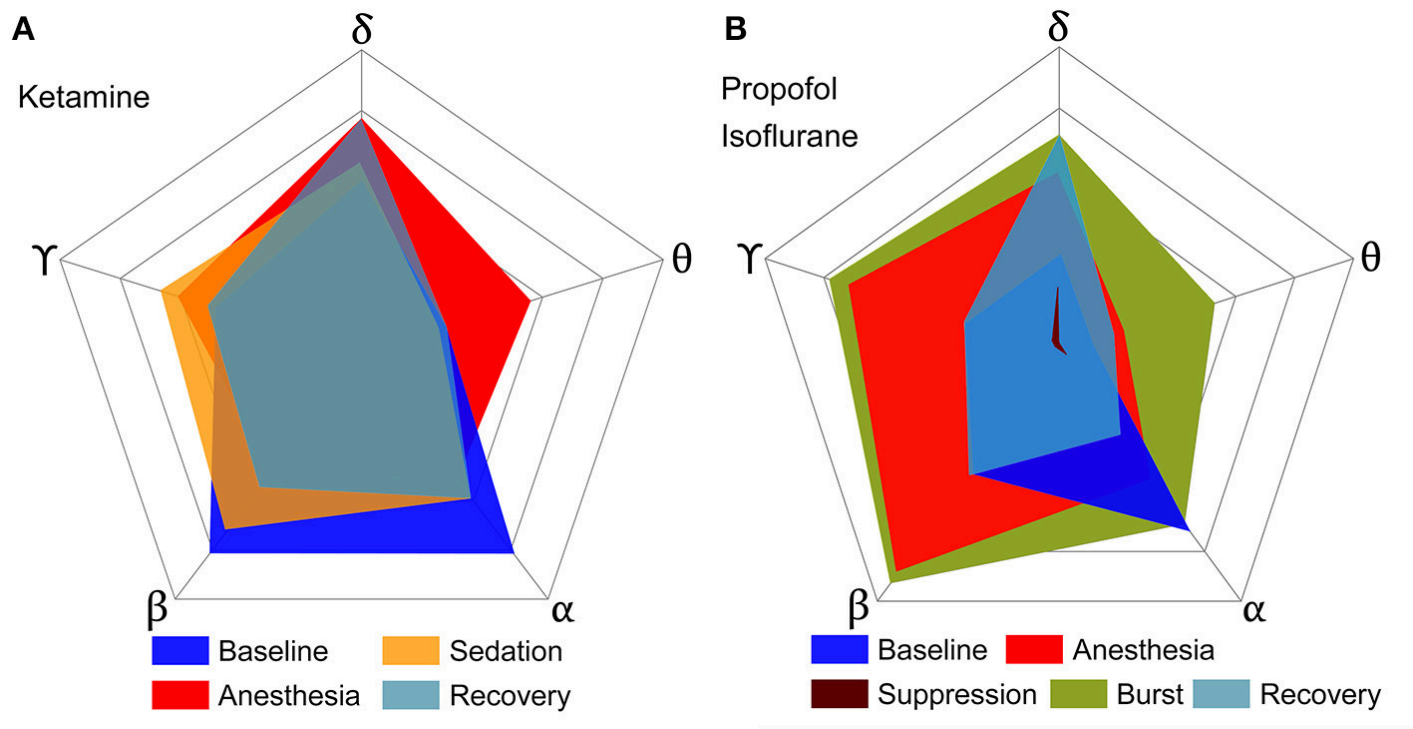
Configurations of $\bar{\Phi }$ over five frequency bands of EEG for various states of consciousness induced by **(A)** ketamine and **(B)** propofol followed by isoflurane. Each axis represents a frequency band of EEG, and different colors represent different states. **(A,B)** show the results of two independent experiments, and the outmost pentagon represents the 0.4 and 0.6 of $\bar{\Phi },$ respectively. The square root of the area is equal to the sum of $\bar{\Phi }$ for each state (significance levels in Supplementary Table 1). The anesthetics in both experiments do not simply reduce or enhance the $\bar{\Phi }s$ of all frequency bands, but rather demonstrate complex configurations of $\bar{\Phi }s$ depending on frequency. Only the suppression period has significant decreases of $\bar{\Phi }s$ for all frequency bands.

To determine the significance of $\bar{\Phi }$ derived from the EEG as a correlate of consciousness, it is necessary to examine its dependence on the spectral components, which are often used to characterize specific states of consciousness. To this end, we calculated the $\bar{\Phi }$ from EEG data filtered to five frequency bands (delta, theta, alpha, beta, and gamma) in various states (baseline, sedated, non-responsive, non-responsive with burst and suppression activities, and recovery state). After anesthesia, ketamine increased the relative powers of delta, theta, and gamma bands, and decreased the relative power of alpha and beta bands. On the contrary, propofol and isoflurane increased the beta power and decreased the gamma power. Figure 2 illustrates the effects of (1) ketamine, and (2) propofol followed by isoflurane on individual $\bar{\Phi }$ values in each frequency band. The anesthetics do not simply reduce or enhance the $\bar{\Phi }$ values of all frequency bands after loss of consciousness, but rather demonstrate complex configurations of $\bar{\Phi }s$ depending on the frequency. Specifically, the $\bar{\Phi }$ of delta, theta, and gamma bands significantly increased, whereas $\bar{\Phi }$ in the alpha band decreased with both anesthetic regimens (significance levels in Supplementary Table 1). During deep anesthesia, $\bar{\Phi }$ during the burst period was higher than baseline (green area in Figure 2B), whereas $\bar{\Phi }$ of the suppression period was near zero (brown area in Figure 2B). The change of $\bar{\Phi }$ over the five frequency bands was variable across states (red areas in Figures 2A,B), demonstrating that surrogates of integrated information in specific frequency bands can significantly increase even during general anesthesia. The significant changes in $\bar{\Phi }$, EEG power, average node degree, and number of modular network structures between baseline and anesthesia are compared in Table 2. Of the four parameters examined, only $\bar{\Phi }$ showed significant and consistent changes in all frequency bands (significance levels in Supplementary Table 2). Furthermore, only $\bar{\Phi }$ during the suppression period in deep anesthesia had values approaching zero in all frequency bands, irrespective of variable EEG powers and network connectivity.

**Table 2:** The changes of power, connectivity, modularity, and Φ of the conventional frequency bands of EEG after anesthesia with ketamine and propofol.

** 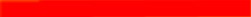 Ketamine**	**Delta**	**Theta**	**Alpha**	**Beta**	**Gamma**
** 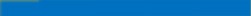 Propofal**	**(0.1–4 Hz)**	**(4–8 Hz)**	**(8–13 Hz)**	**(13–25 Hz)**	**(25–45 Hz)**
Relative power of spectrogram	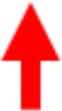	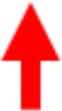	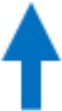	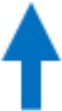 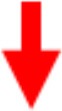	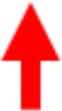 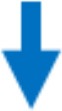
Integrated information (${\bar{\Phi }}_{\text{AR}}$)	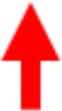 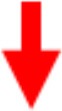	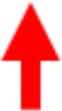 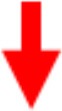	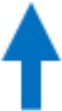 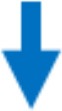	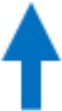 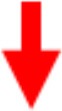	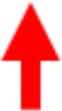 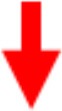
Average degree (weighted PLI network)	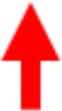 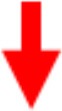		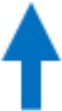 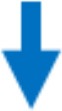		
Number of modules (modular structure)	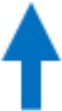	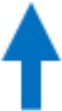	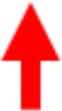 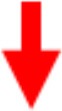		
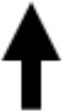 Increase in anesthetized state 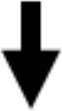 Decrease in anesthetized state					

*Note that, for power, arrows represent overall values and that significant regional differences could still be observed. The red and blue arrows denote the significant changes (significance levels in Supplementary Table 2)*.

### Relationship between $\bar{\Phi }$ and functional brain network structure

**Figure 3:**
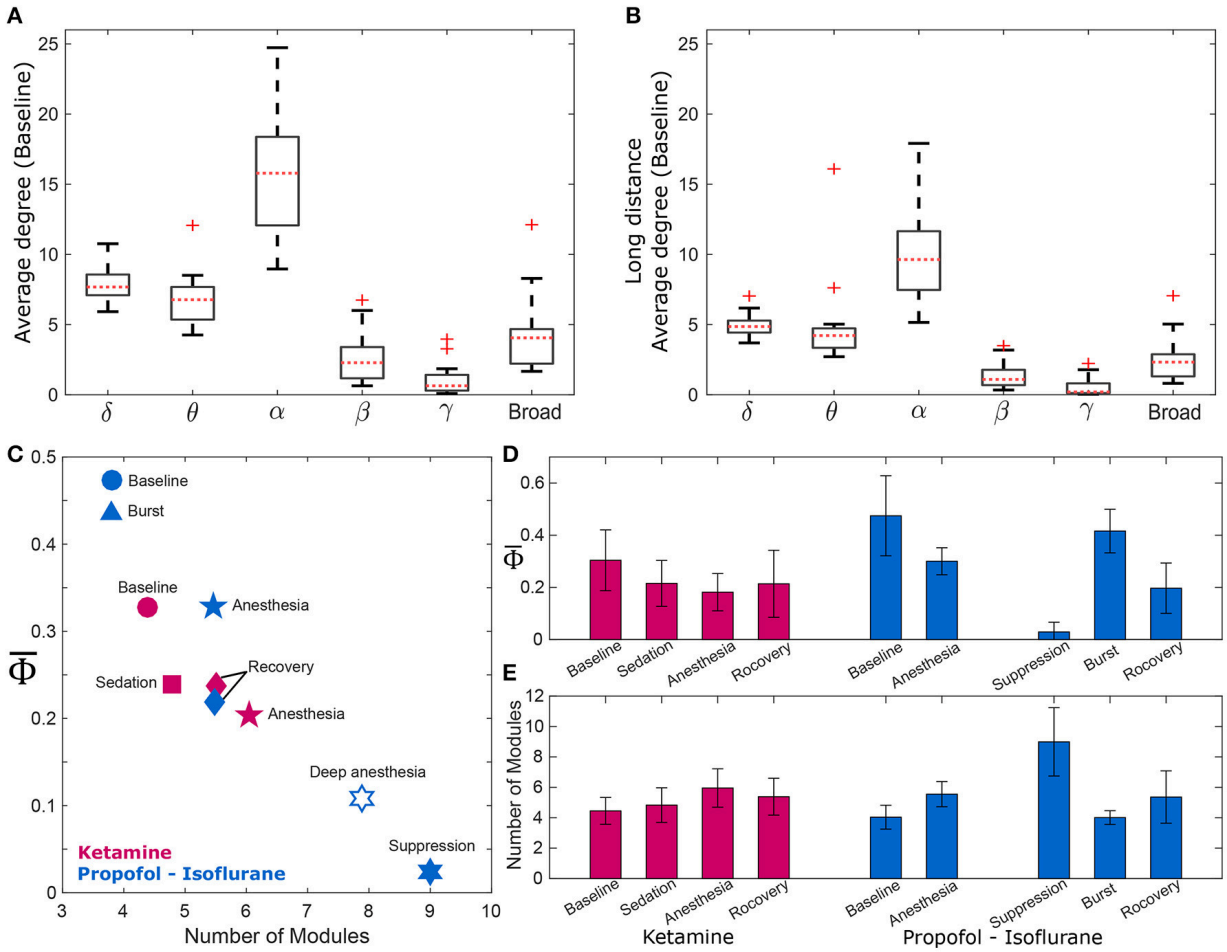
$\bar{\Phi }$ and the modularity of the EEG network. Comparison of average degrees for **(A)** all connected nodes in baseline state, and **(B)** long-distance connections across six frequency bands (delta, theta, alpha, beta, gamma, and broad band). The alpha band shows significantly higher degrees for both cases (*p* < 0.01). The long-distance connections were defined as nodes that have distances longer than 9 cm in the actual physical distances (1.8–18 cm) between all EEG electrodes. For the alpha band, **(C)** strong negative correlation between $\bar{\Phi }$ and the number of modules of wPLI network was demonstrated, and **(D,E)** significant changes of $\bar{\Phi }$ and the number of modules in wPLI network across states in the two experiments. The $\bar{\Phi }$ and the number of modules significantly change across states. (significance levels in Supplementary Table 3). The other frequency bands were also tested (Supplementary Figure 3).

To find a relationship between $\bar{\Phi }$ and functional brain network structure, we focused on the alpha band. Functional brain network structure is critical to integrate spatially distributed information and dense functional connections may facilitate information integration. We observed that the alpha band had a significantly larger average degree and long range connectivity compared with other frequency bands (Figures 3A,B). Thus, we assumed for the current analysis that the $\bar{\Phi }$ and the network modularity of the alpha band could best reflect the relationship between integrated information and functional brain network structure.

There was a strong negative correlation between $\bar{\Phi }$ and the number of modules (*R* = −0.87, *p* = 0.0045), which is shown in Figure 3C. As expected, $\bar{\Phi }$ was largest in baseline (blue and red circles) and lowest in deep anesthesia (unfilled star). The number of modules was lower during baseline consciousness and higher during deep anesthesia. As we separated isoflurane anesthesia into burst and suppression periods that extended over 21% and 79% of the time, the suppression period had the lowest $\bar{\Phi }$ and the largest number of modules. Conversely, the burst period had the largest $\bar{\Phi }$ and the smallest number of modules. $\bar{\Phi }$ decreased and the number of modules increased during ketamine anesthesia as well. However, these variables failed to regain their baseline level after recovery of consciousness in both anesthesia protocols, potentially suggesting incomplete elimination of the anesthetic drugs from the brain at the time of EEG measurement (in Figures 3D,E). The relationship between $\bar{\Phi }$ and the number of modules was also tested with the other frequency bands (Supplementary Figure 3). Interestingly, only specific frequency bands (theta, alpha, and beta) showed significant correlations between $\bar{\Phi }$ and the number of modules (Spearman correlation coefficients; *R* = −0.78, −0.87, and −0.78, *p* < 0.05, respectively). Analysis of theta, alpha, and beta bands demonstrated a higher number of modules in association with smaller $\bar{\Phi }$. In other words, the assessment of integrated information of theta, alpha, and beta bands is associated with global network properties, whereas the integrated information of delta and gamma bands does not appear to be influenced by functional integration and segregation of the brain network.

### Topographic structures of ${\bar{\Phi }}_{R}$ and EEG connectivity across states of human consciousness

**Figure 4:**
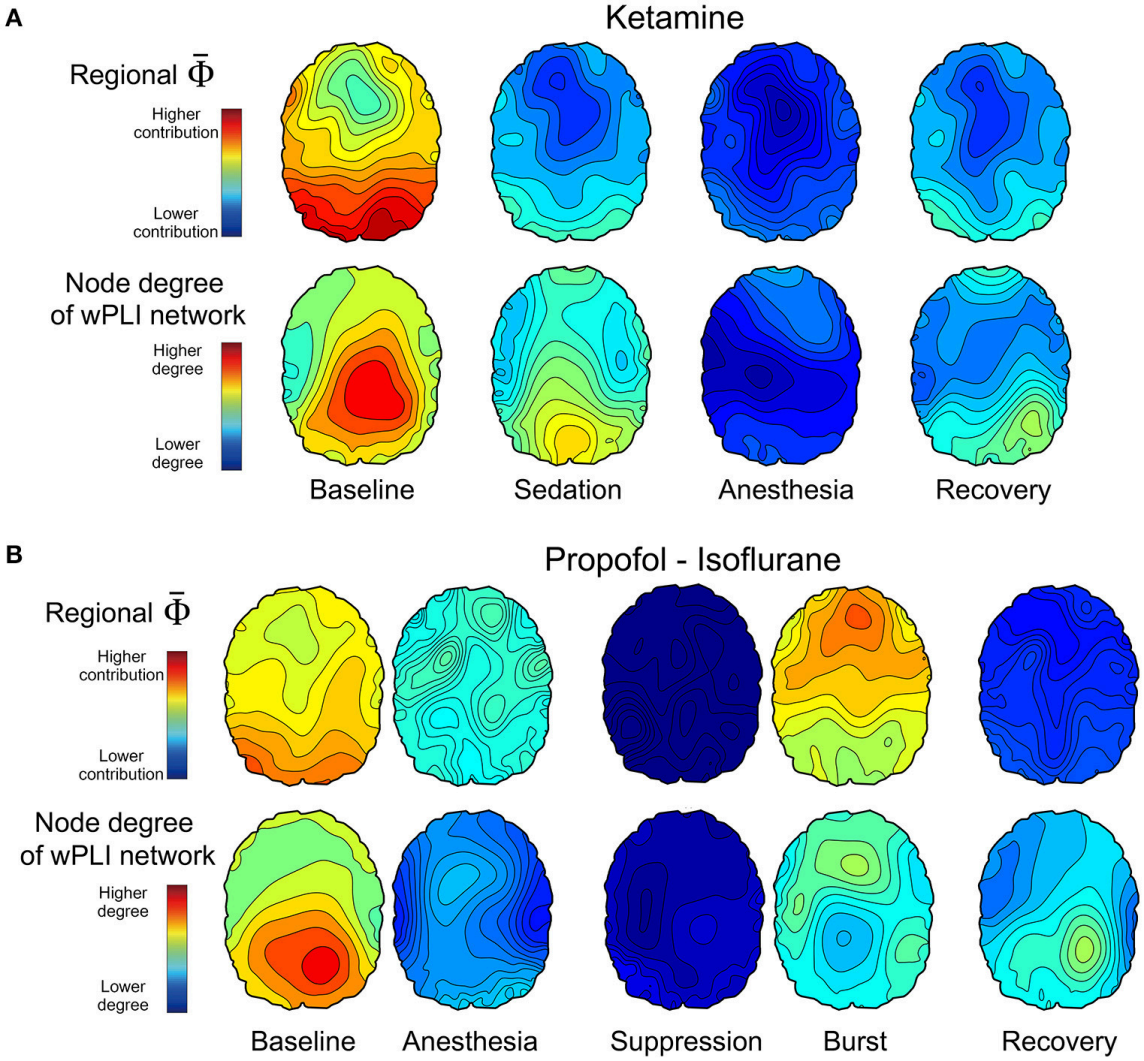
Topographic structures of ${\bar{\Phi }}_{R}$ and EEG connectivity of the alpha band across states of consciousness in the two anesthetic experiments of **(A)** ketamine, and **(B)** propofol followed by isoflurane. The first and second row in **(A,B)** present the topography structure of ${\bar{\Phi }}_{R}$ and node degree of 96 EEG channels, respectively, which is averaged over all subjects across states. The topographical structures and the overall strengths represent various states of consciousness. For example, higher ${\bar{\Phi }}_{R}$ and node degree of the posterior region in the baseline state is disrupted in the anesthetized state and restored partly with recovery of responsiveness. The scales of ${\bar{\Phi }}_{R}$ and node degree topographies are the same among the states in each experiment, but different between the two experiments. The topographies of node degree and ${\bar{\Phi }}_{R}$ for the other frequency bands are presented in Supplementary Figure 4.

We estimated the contribution of a single EEG channel on $\bar{\Phi }$, which we term ${\bar{\Phi }}_{R}$, and compared this with the node degree of functional connectivity network in each state. We hypothesized that network nodes with a high density of functional connections, i.e., network hubs, would make a higher contribution to the whole system's integrated information and that a disruption of hub connectivity during anesthesia would be accompanied by a decrease in integrated information, that is, a decrease in $\bar{\Phi }$. Figure 4 demonstrates that the topographic structures of node degree and ${\bar{\Phi }}_{R}$ of the alpha band were similar to each other although the match was not exact. The spatial patterns of node degree and ${\bar{\Phi }}_{R}$ were also state-specific. In the baseline condition, node degree and ${\bar{\Phi }}_{R}$ were higher in the posterior regions but this distribution diminished in anesthesia. The topographic structures during recovery were similar to (Ketamine, Figure 4A) or even below (Propofol-Isoflurane, Figure 4B) those in subanesthetic sedation and did not approximate the corresponding levels in the awake baseline. Interestingly, during the burst periods of isoflurane anesthesia, the ${\bar{\Phi }}_{R}$ substantially increased in the frontal area (Figure 4B). This suggests that the spatial information integration during bursting may be qualitatively different from that in the conscious baseline, despite a similar $\bar{\Phi }$ (Figure 3B). The topographies of node degree and ${\bar{\Phi }}_{R}$ for the other frequency bands (delta, theta, beta, gamma, broad band) are presented in Supplementary Figure 4.

### Multi-dimensional parameter space based on ${\bar{\Phi }}_{R}$ and EEG connectivity

**Figure 5:**
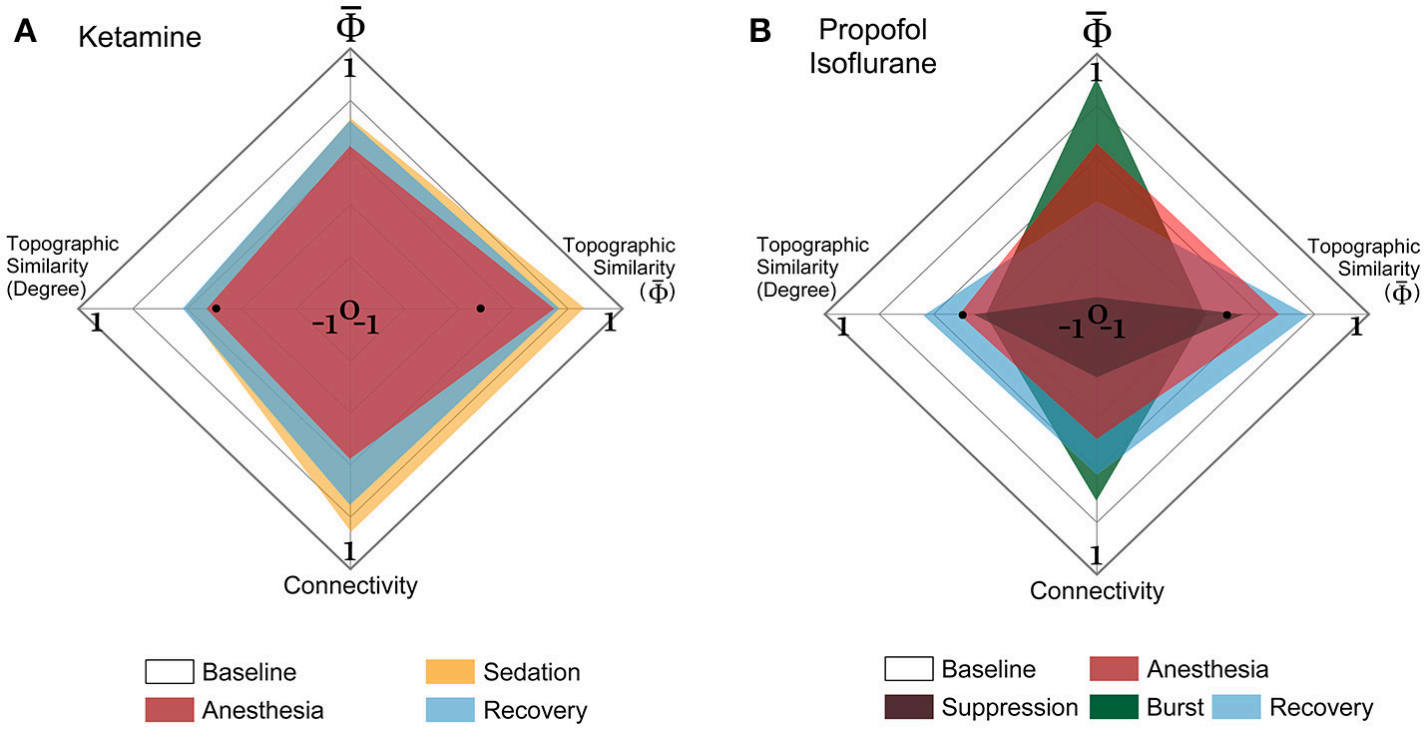
Multi-dimensional parameter space based on EEG connectivity and ${\bar{\Phi }}_{R}$ in the alpha band. The various states induced by **(A)** ketamine and **(B)** propofol-isoflurane are represented in multi-dimensional parameter spaces, which consist of the relative $\bar{\Phi }$ and relative connectivity as well as the topographic similarities of ${\bar{\Phi }}_{R}$ and node degree. Variables are all normalized to the baseline. In the 4-dimensional parameter space, the scales are (−1 to 1) in the horizontal axes for both topographic similarities and (0 to 1) in the vertical axes for $\bar{\Phi }$ and connectivity. Ketamine and propofol-isoflurane produce different states of consciousness, which are represented as characteristic regional shapes in the parameter space (significance levels in Supplementary Table 4).

The EEG connectivity and ${\bar{\Phi }}_{R}$ complement each other and represent different aspects of a brain state. By definition, the EEG connectivity reflects statistical similarity between activities across brain regions and the ${\bar{\Phi }}_{R}$ is intended to reflect the amount of integrated information, possibly through the functional connectivity. The strengths and the topographic similarities of EEG connectivity and ${\bar{\Phi }}_{R}$ change significantly during the anesthetized state. In Figure 5, the multi-dimensional parameter space consists of the strength and the topographic similarity of EEG connectivity and of ${\bar{\Phi }}_{R}$, differentiating the overlapped states in terms of the strength (for instance, baseline and burst state in deep anesthesia in Figure 5B). Figures 5A,B illustrate the multi-dimensional parameter spaces of the alpha band for the two experiments with ketamine and propofol-isoflurane. The size and shape of the area represent the profile of the strength and topological similarity of a state. The significance levels of the comparisons between states at each axis are presented in Supplementary Table 4. In the ketamine experiment (Figure 5A), the topographic similarities of node degree and ${\bar{\Phi }}_{R}$ decrease significantly during anesthesia (*p* < 0.01). Sedation has a significantly higher node degree and topographic similarity of ${\bar{\Phi }}_{R}$ than the recovery state (*p* < 0.05), which means that along certain dimensions the sedated state is closer to the baseline. In the propofol-isoflurane experiment (Figure 5B), anesthesia with propofol and suppression with isoflurane induces significant decreases in all dimensions (*p* < 0.05) compared to the baseline. The burst period with isoflurane had similar $\bar{\Phi }$ and node degree compared with the baseline. However, the topological similarity of node degree and ${\bar{\Phi }}_{R}$ demonstrate consistent and significant changes across all frequency bands during all anesthetic states in both experiments (*p* < 0.01), whereas the node degree and $\bar{\Phi }$ show state- and anesthetic-specific responses. The multi-dimensional parameter space of the other frequency bands (delta, theta, beta, gamma, and broad band) are presented in Supplementary Figures 5, 6.

## Discussion

### Summary of the findings

According to IIT, the level of consciousness is related to the system's functional differentiation and overall integration, mathematically expressed by Φ. The physical quantity and spatiotemporal grain necessary to measure Φ empirically is unclear. To test if there was a relationship between Φ estimated from EEG and various states of human consciousness, we modulated the state of healthy human volunteers with different anesthetics in two independent experiments. We introduced a method to estimate the relative change of Φ across states of consciousness referenced to the baseline, rather than calculating the precise Φ of a state. The method is based on estimating the mean integrated information $\bar{\Phi }$ of many small sample units of randomly selected EEG channels. In the analysis, we found that $\bar{\Phi }$ is not simply reduced along with the loss of consciousness, defined as non-responsiveness during general anesthesia. Different anesthetics gave rise to distinctive profiles of spectral power and connectivity depending on the frequency. The comparison between power, connectivity, and $\bar{\Phi }$ before and after general anesthesia demonstrated that increased or decreased power and connectivity of EEGs tend to increase or decrease $\bar{\Phi }$, respectively, independent of frequency bands (Table 2). Only the alpha band showed consistently decreased $\bar{\Phi }$ in both anesthetic experiments and only the suppression period in the deeply anesthetized state was associated with near zero $\bar{\Phi }$ in all frequency bands. The results suggest that the state transition from baseline (larger $\bar{\Phi }$) to the deeply anesthetized state (near zero $\bar{\Phi }$) is not monotonic in terms of increase or decrease of integrated information, but variable across states and frequency bands (in Figure 2). Moreover, the large $\bar{\Phi }s$ of some frequency bands during general anesthesia do not match our prediction based on IIT. The reason for the relatively high $\bar{\Phi }$s of some frequency bands during general anesthesia and relatively low $\bar{\Phi }s$ during the recovery state is unclear. There are inherent limitations to the data acquisition method, network reconstruction method, and analytic method that could potentially distort $\bar{\Phi }$. It is also possible that the level of $\bar{\Phi }$ may not fully reflect the level of consciousness. The structure of integrated information in addition to the level of $\bar{\Phi }$ might be of importance. This possibility motivated us to investigate the topographic structure of ${\bar{\Phi }}_{R}$ to represent the various states of consciousness.

### Relationship between network structure and $\bar{\Phi }$

The alpha band was associated with the highest node degree and long-range functional connectivity (Figures 3A,B). We hypothesized that the alpha band may play an important role for integrating information in the brain network, thus, there may be a relationship between $\bar{\Phi }$ and alpha connectivity. The modular structure of network connectivity was analyzed because of its presumed relevance for information integration (Tononi, 2012; Oizumi et al., 2014). We found a significant correlation between the modular structure of functional networks and $\bar{\Phi }$ consistent with the idea that a high number of modules (greater modularity) in a network is incompatible with global information integration. Interestingly, we found that the correlation between $\bar{\Phi }$ and the number of modules is frequency specific: the theta, alpha, and beta bands demonstrated significant (and negative) correlations, whereas the delta and gamma bands did not (Supplementary Figure 3). The results imply that the integrated information in theta, alpha, and beta bands is associated with the global network property (modularity), whereas the (increased or decreased) integrated information in delta and gamma bands is less affected by the modularity in the functional brain network.

### Burst vs. EEG silence

When data from EEG burst and suppression periods were analyzed separately, the Φ of the EEG bursts is similar to or even larger than wakeful consciousness. This could suggest a similar or higher level of consciousness during the burst period in deep anesthesia. However, other evidence suggests alternative interpretations. First, the topographical structures of node degree and ${\bar{\Phi }}_{R}$ are significantly different between burst and wakefulness. For instance, ${\bar{\Phi }}_{R}$ for the alpha band are higher in the posterior part of the brain during wakefulness and higher in the frontal ${\bar{\Phi }}_{R}$ during bursts. This implies that the integrated information during bursting is qualitatively different from that in the conscious baseline, despite a similar $\bar{\Phi }$. Second, Mudrik et al. suggested that information integration can occur without conscious awareness, as the integration scope is limited to smaller integration windows or to simpler associations (Mudrik et al., 2016). This raises the question of whether Φ specifically measures *conscious* integration or also *unconscious* integration–a question that does not yet have a definitive answer. Third, another potentially important difference is that during wakeful consciousness both Φ and modularity are temporally persistent whereas during burst suppression they are temporally fragmented. In our data, the burst periods occupied about 20% of deep anesthesia and each burst period lasted for a few seconds. Bursts are hypersynchronous states featuring high integration but low differentiation (Li et al., 2013). They facilitate spatial integration (low modularity) but disrupt temporal integration. It is likely that both temporal and spatial integration of distributed information are necessary for the maintenance of wakeful consciousness. Otherwise, conscious brain states may not recover during the short bursts or, even if they do, overt responsiveness may not emerge within the limited duration of the bursts available for integration.

Obviously, the separation of burst and suppression periods into independent states may not be clinically relevant. Without such separation, the $\bar{\Phi }$ during deep anesthesia had the lowest $\bar{\Phi }$, which is consistent with the general prediction of IIT. However, this is unsatisfactory based on theoretical grounds: time-averaging over burst suppression conflates the segments of high EEG activity and absent activity, with electrocortical silence contributing zero to EEG power, connectivity, Φ, etc. This means that during burst suppression, the Φ, modularity and other related variables are only meaningful if estimated separately for active and inactive electrocortical states.

### Multi-dimensional representations of consciousness

The multi-dimensional parameter representation (Figure 5 and Supplementary Figures 5, 6) revealed how ketamine and propofol altered the strength and the topographic similarity of ${\bar{\Phi }}_{R}$ and connectivity in distinct ways (Bayne et al., 2016). Anesthetized states with ketamine and propofol as well as deep anesthesia with isoflurane were all accompanied by a disruption of topographic similarity of connectivity and ${\bar{\Phi }}_{R}$. Notably, the multi-dimensional parameter space clearly differentiated the burst period from the baseline, with differences of topographic connectivity and ${\bar{\Phi }}_{R}$ compared with the baseline.

The recovery of responsiveness following anesthesia in the absence of a full recovery of integrated information implies that there may be a threshold of integration above which subjects regain purposeful responsiveness (Tononi, 2008; Tononi and Koch, 2015; Barbosa, 2017). The differential values are consistent with what is likely a richer phenomenology at baseline compared to just after emergence from anesthesia. The multidimensional representation of parameters including network connectivity and ${\bar{\Phi }}_{R}$ of the brain promises to be a useful approach to further differentiate the level and contents of consciousness at different behaviorally defined states.

### Limitations of estimating $\bar{\Phi }$ from EEG

In this study, Barrett and Seth's algorithm, ${\tilde{\Phi }}_{AR}$, (Barrett and Seth, 2011) was used to estimate Φ from the high-density EEG recording during various states of consciousness. However, Barrett's algorithm contains potential problems. If EEG is contaminated with highly correlated noise, ${\tilde{\Phi }}_{AR}$ could fail to satisfy the theoretical upper boundary of Φ. In a recent paper, Oizumi et al. pointed out the theoretical problem of Barrett's algorithms (Oizumi et al., 2016a), which do not satisfy the theoretical upper and lower boundaries of Φ, i.e., the amount of integrated information should not be negative and never exceed the information generated by the whole system. Our random EEG channel selection circumvents this potential problem of ${\tilde{\Phi }}_{AR}$ by reducing the correlated noise, mostly due to volume conduction, among spatially proximal EEG channels. In addition, the Φ of surrogate data was subtracted to reduce the spurious Φ due to the EEG frequency profiles and various volume conduction effects of diverse states during anesthesia. Moreover, considering the fundamental limitation of scalp EEG for recording neuronal population activity in different experimental conditions, we only interpreted the relative changes of $\bar{\Phi }$ across states referenced to the baseline. Furthermore, the near-zero $\bar{\Phi }$ of the suppression period suggests a negligible effect of correlated noise in our EEG data.

Oizumi et al. attempted to solve the theoretical problem of calculating Φ from empirical time series with a new approach called “mismatched decoding,” but the method is subject to the limitation of a Gaussian assumption (Oizumi et al., 2016a). In practice, the errors resulting from a Gaussian assumption would be difficult to correct if the EEG signals of diverse states of consciousness have non-Gaussian distributions. In order to examine the effect of the Gaussian assumption, we calculated ${\tilde{\Phi }}_{E}$, Barrett's other Φ measure using a Gaussian assumption, and Φ^*^ from Oizumi et al. Both measures with a Gaussian assumption showed similar patterns to each other across the five states, which are also comparable with ${\tilde{\Phi }}_{AR}$. However, the Φ^*^ of the suppression period was much higher than zero (in Supplementary Figure 7).

## Conclusion

This study introduced a novel and practical method to estimate Φ from high density EEG and applied it to various states of consciousness altered by general anesthesia induced by ketamine and propofol-isoflurane. The investigation of the EEG properties corresponding to large and small $\bar{\Phi }$ enabled us to infer large-scale network correlates of various states of consciousness. The multi-dimensional parameter space consisting of various EEG-derived measures of connectivity and ${\bar{\Phi }}_{R}$ was efficacious in differentiating various states of consciousness and sub-states during burst and suppression. This practical method could potentially facilitate the empirical application of IIT to various states of consciousness in normal and abnormal brains.

## Author contributions

AH, GM, HK, and UL wrote the manuscript; UL designed research; HK and UL developed the computational methods; HK analyzed data; JL curated data; GM and the ReCCognition Study Group acquired data; All authors reviewed the manuscript.

### Conflict of interest statement

The authors declare that the research was conducted in the absence of any commercial or financial relationships that could be construed as a potential conflict of interest. The reviewer DO and handling Editor declared their shared affiliation, and the handling Editor states that the process nevertheless met the standards of a fair and objective review.
