# Supplementary material for: Estimating the Integrated Information Measure Phi from High-Density Electroencephalography during States of Consciousness in Humans
Source: Front Hum Neurosci. 2018 Feb 16;12:42. doi: 10.3389/fnhum.2018.00042 (PMC5821001; doi:10.3389/fnhum.2018.00042)
Supplement: Supplementary file 1 [file Presentation1.zip › gm, Jan30-Revised-Supplementary-materials-corrected.docx]

Supplementary Material

**Estimating the integrated information measure Phi from high-density electroencephalography during states of consciousness in humans**

Hyoungkyu Kim^1,2^, Anthony G Hudetz^1,2,3,^, Joseph Lee^1^ , George A Mashour^1,2,3^, UnCheol Lee^1,2*^ and the ReCCognition Study Group ^#^

*** Correspondence:** UnCheol Lee: uclee@med.umich.edu

# Supplementary Figures and Tables

## Supplementary Figures


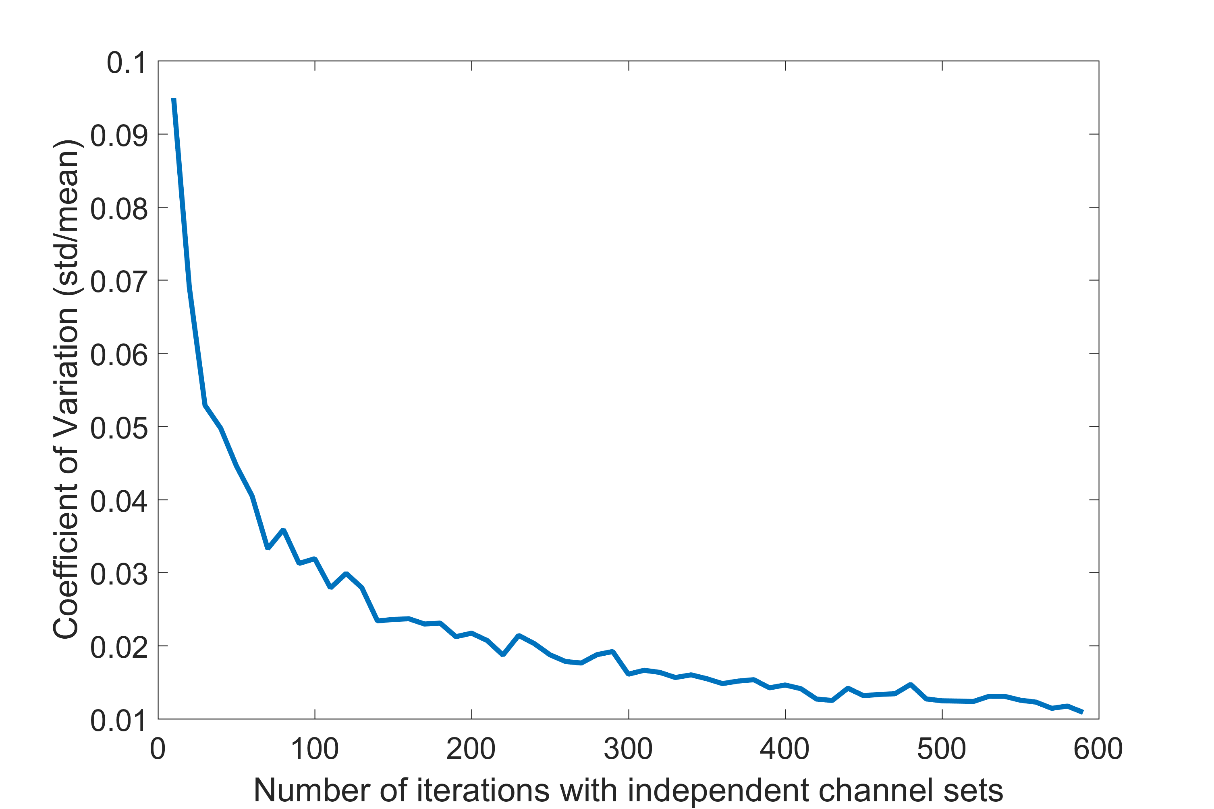


**Supplementary Figure 1.** Coefficient of variation for the iteration number. The coefficient of variance was calculated as the number of iterations increases. The number of sample units and the number of random EEG channels for each sample unit were fixed as 600 and 8, respectively. The coefficient of variance with 600 iterations almost reaches 0.01 with 95% probability.


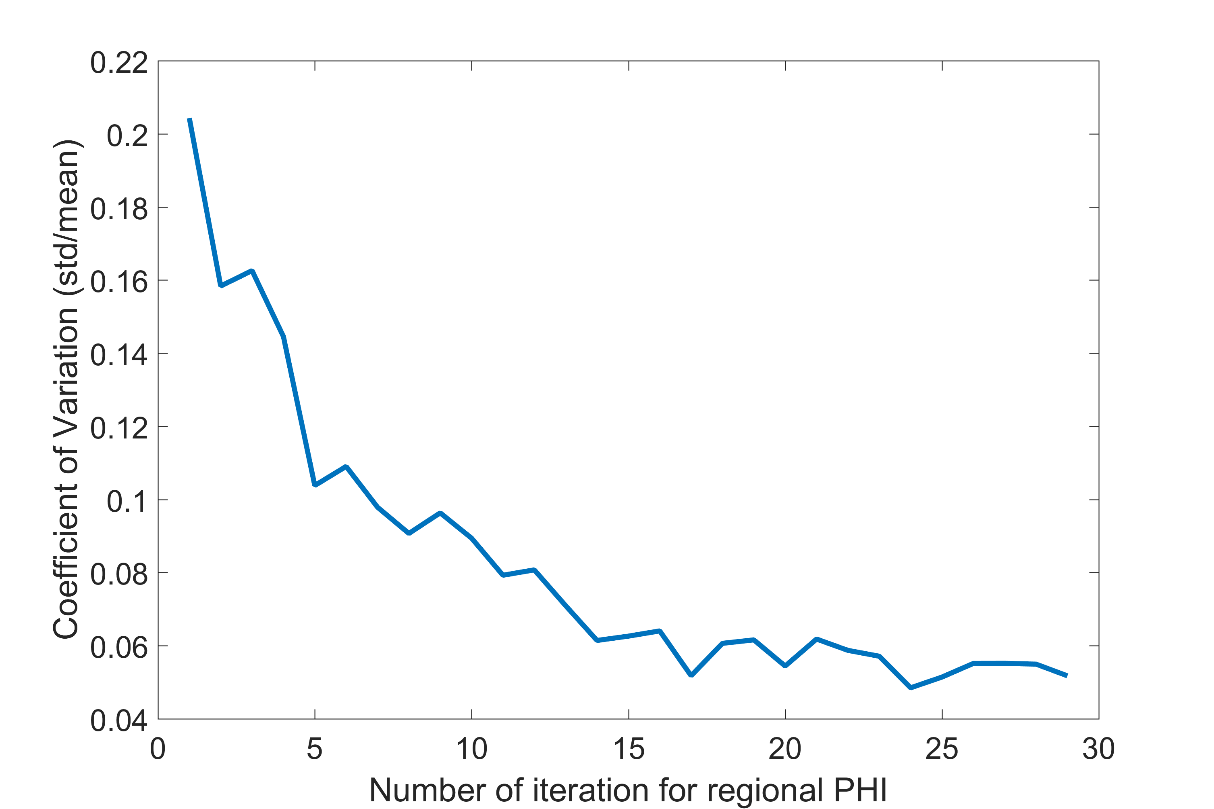


**Supplementary Figure 2.** Coefficient of variation for regional $\bar{\Phi}$. The coefficient of variance was calculated as the number of iterations increases. The number of sample units and the number of random EEG channels for each sample unit were fixed as 30 and 8, respectively. The coefficient of variance with 30 iterations reaches 0.05 with 95% probability.


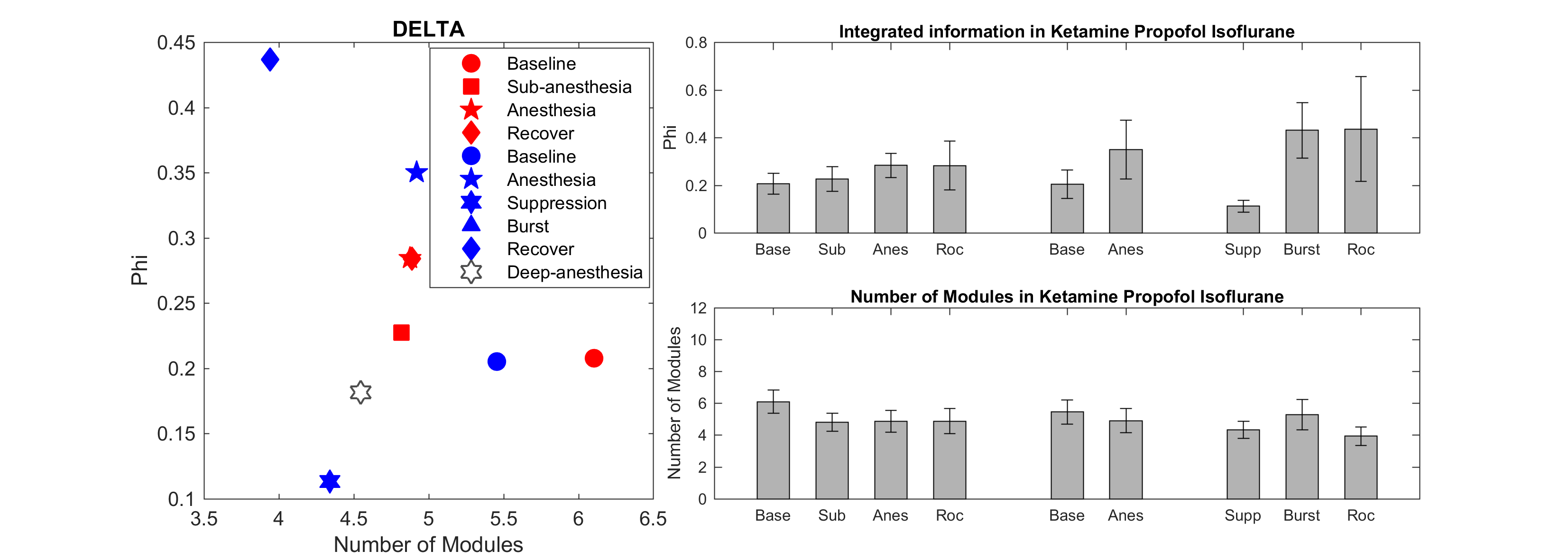

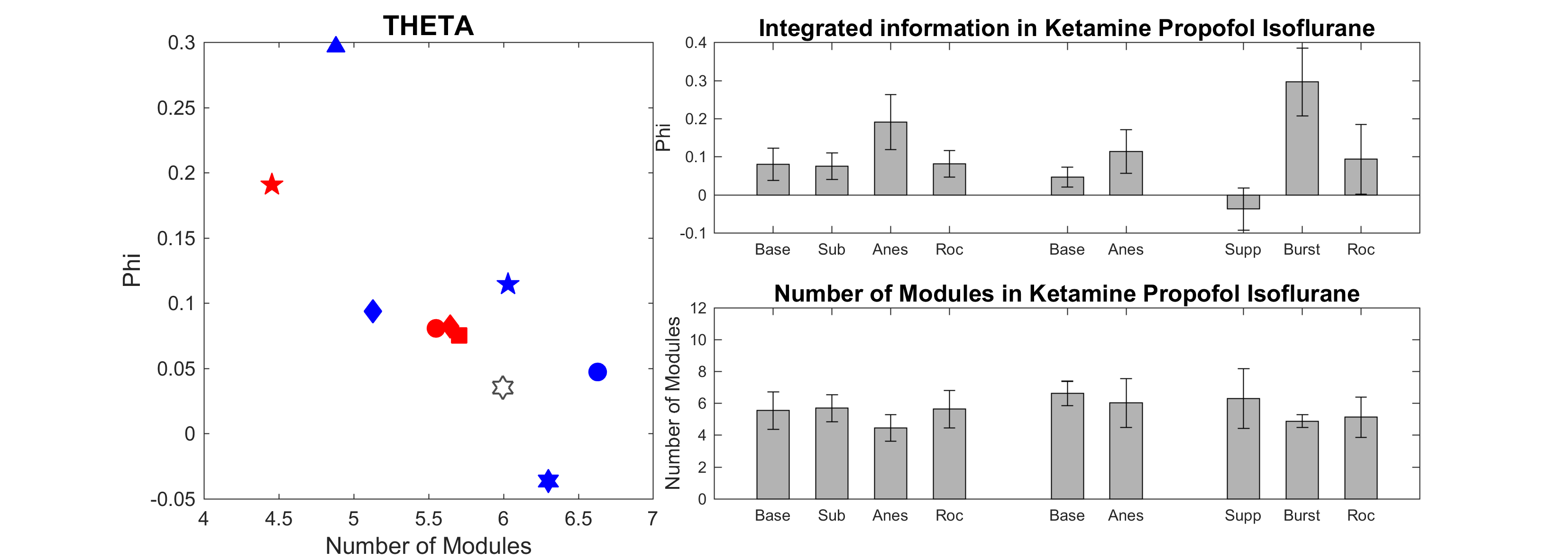


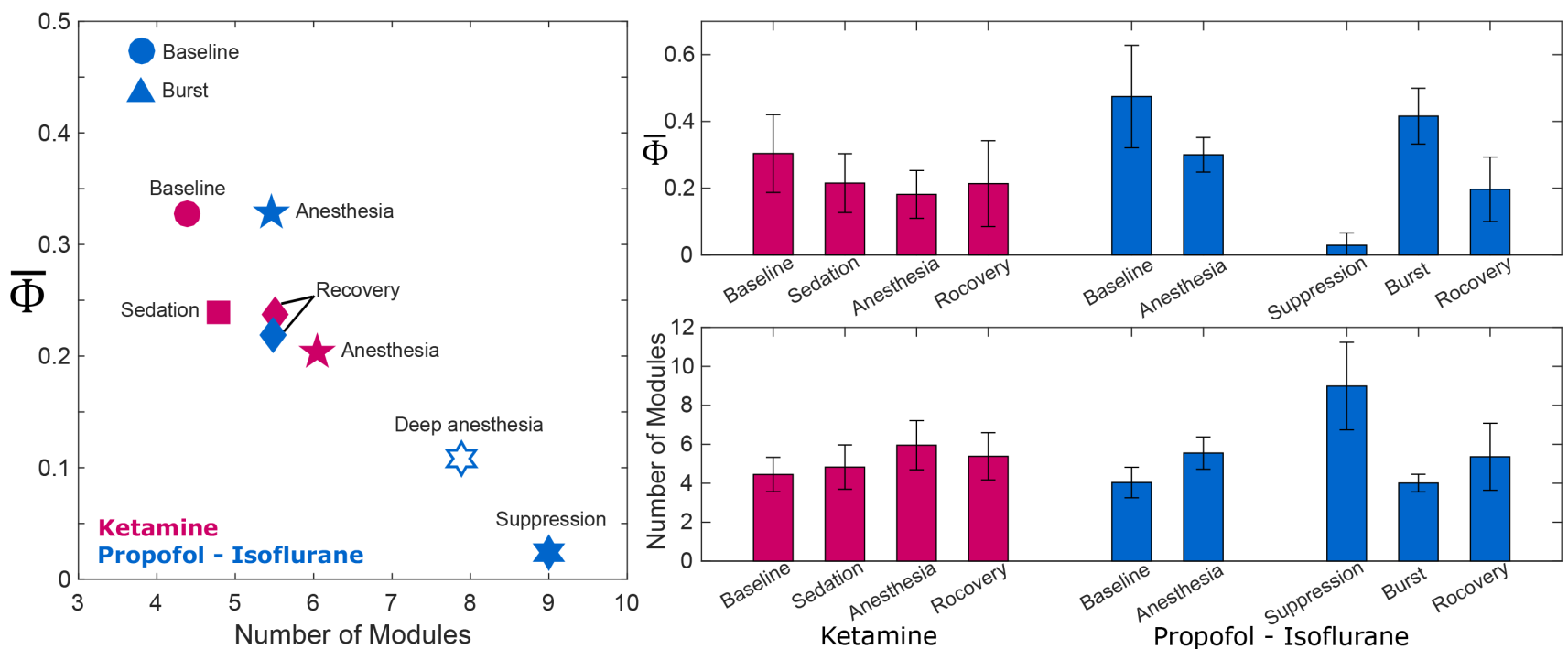


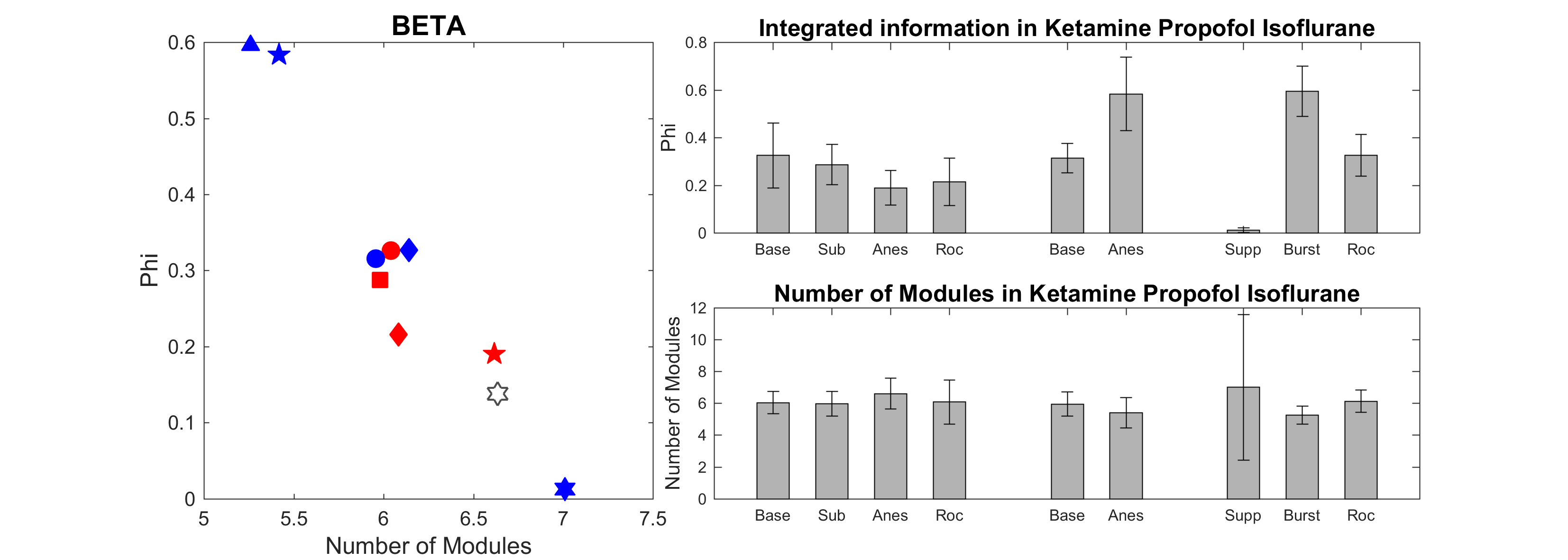

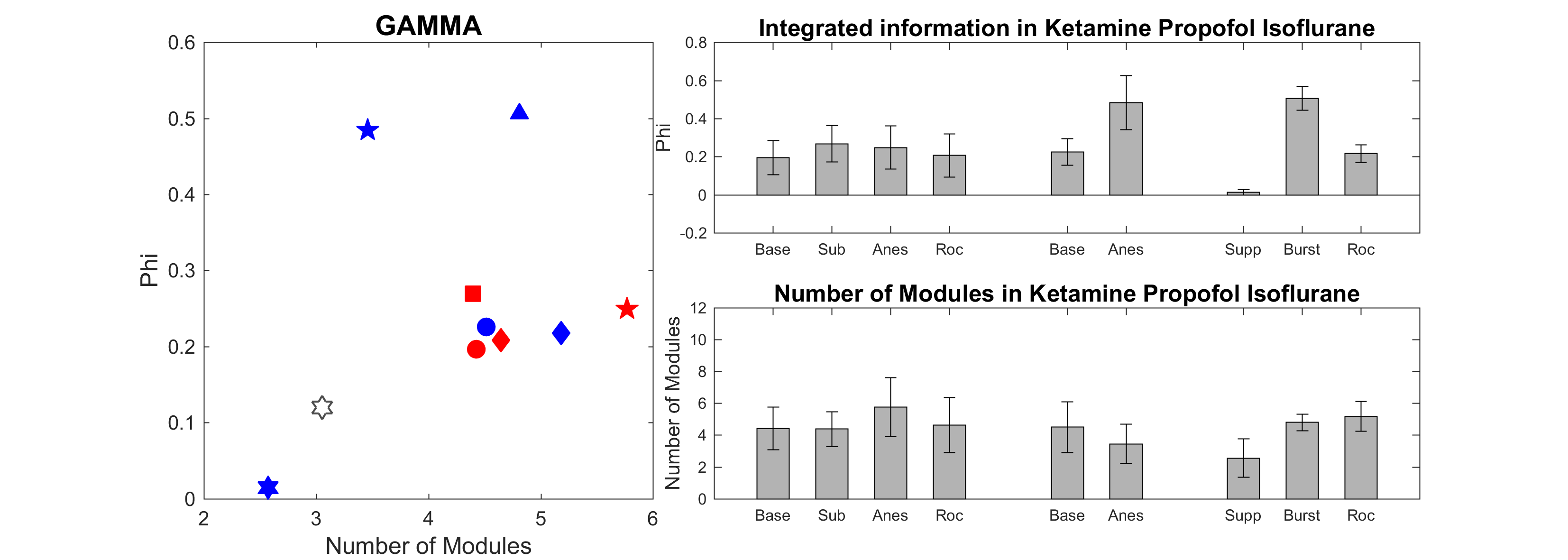

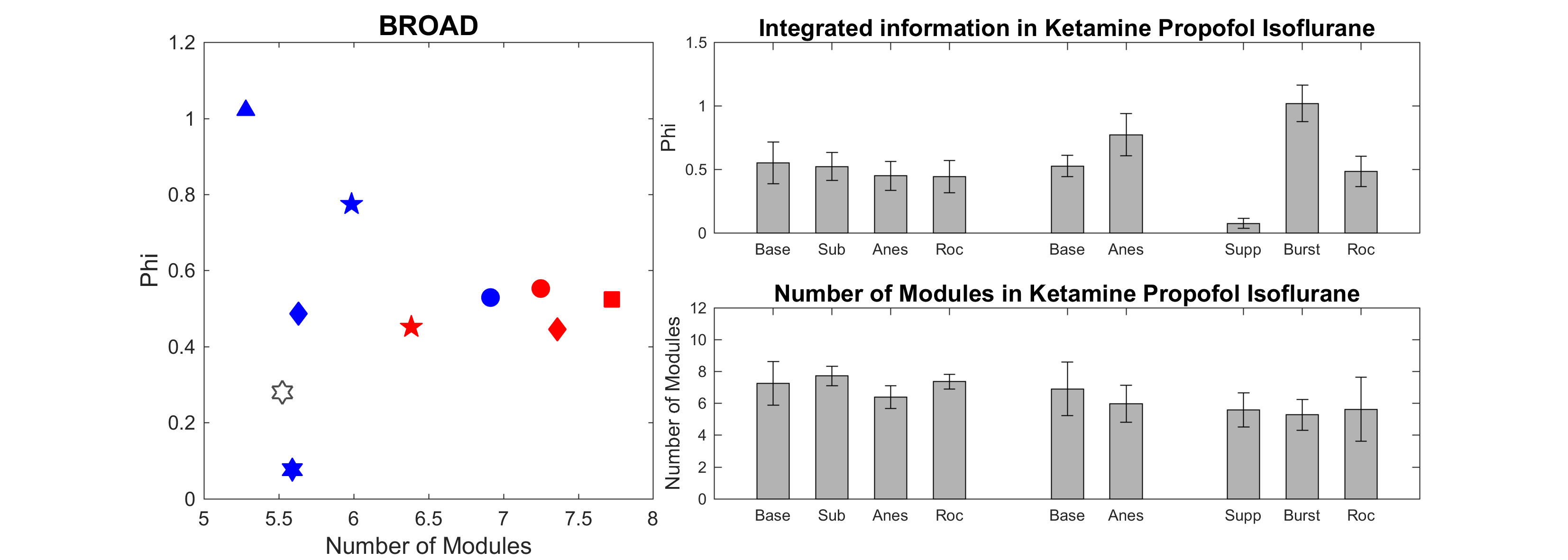


**Supplementary Figure 3.** $\bar{\Phi}$ and the number of modules for the six frequency bands. The significant negative correlation between $\bar{\Phi}$ and the number of modules were observed in the theta, alpha and beta bands (R=-0.78, -0.87, -0.78, p<0.05), and no significant correlation in the other bands (p>0.05).


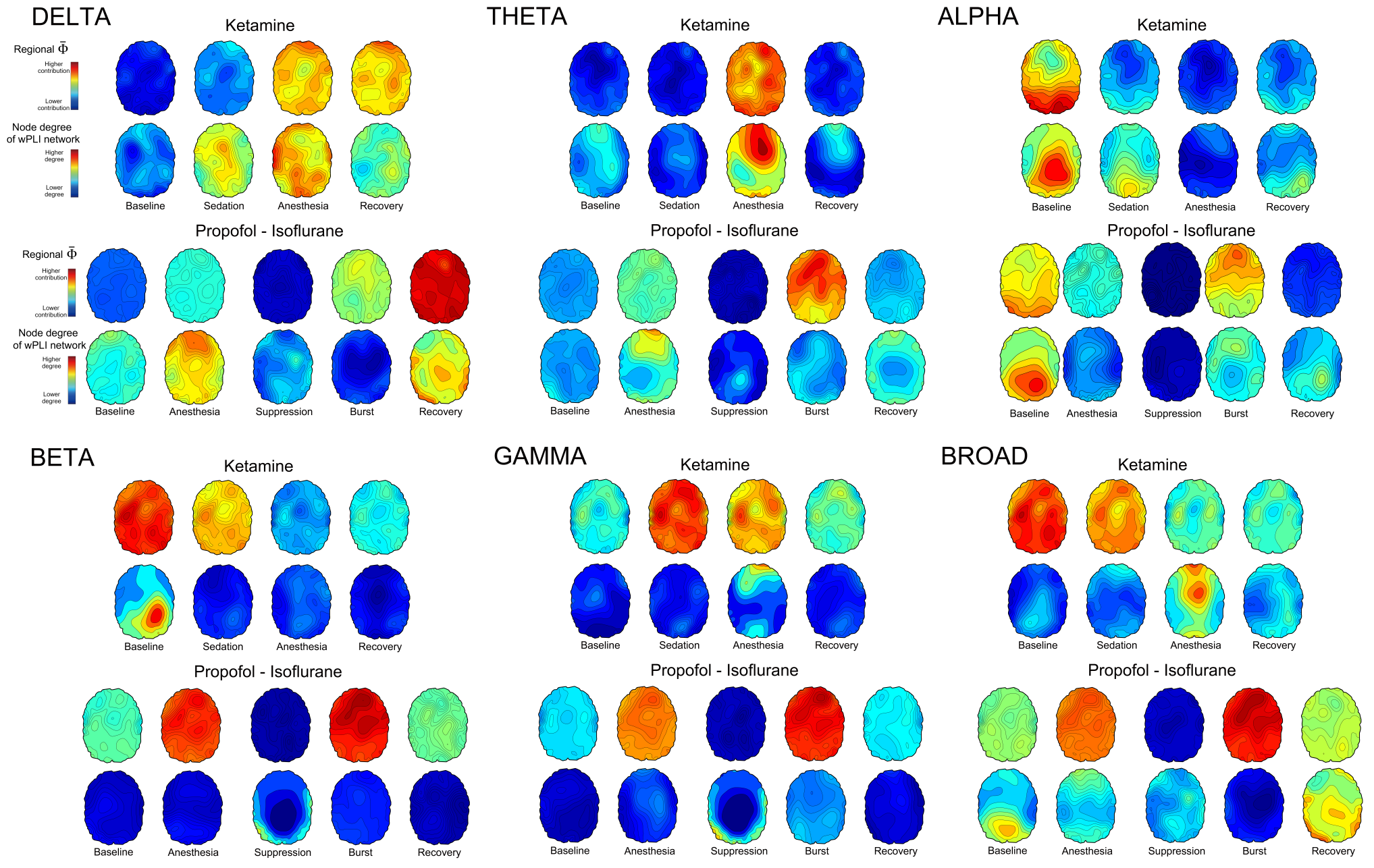


**Supplementary Figure 4.** Regional $\bar{\Phi}$ and EEG connectivity in each frequency band.


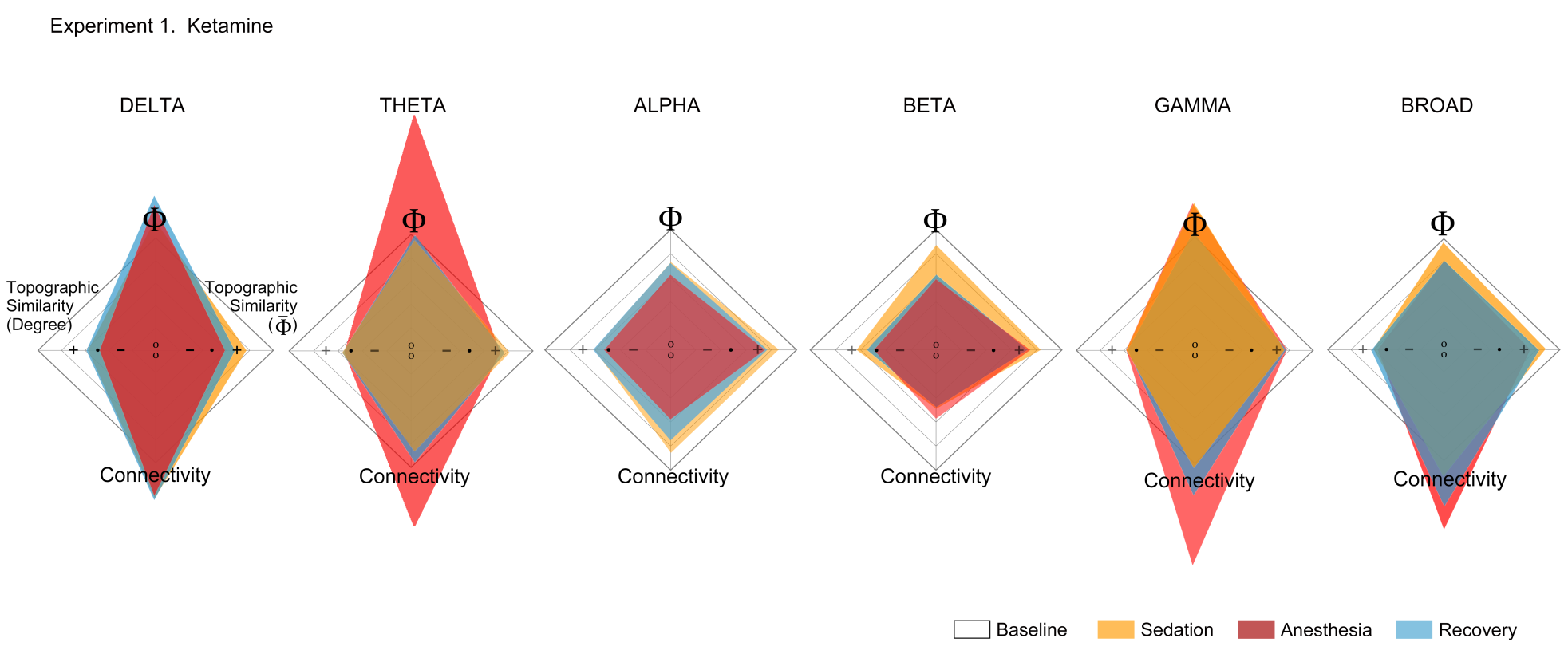


**Supplementary Figure 5.** Multi-dimensional parameter space of ketamine experiment for each frequency band.


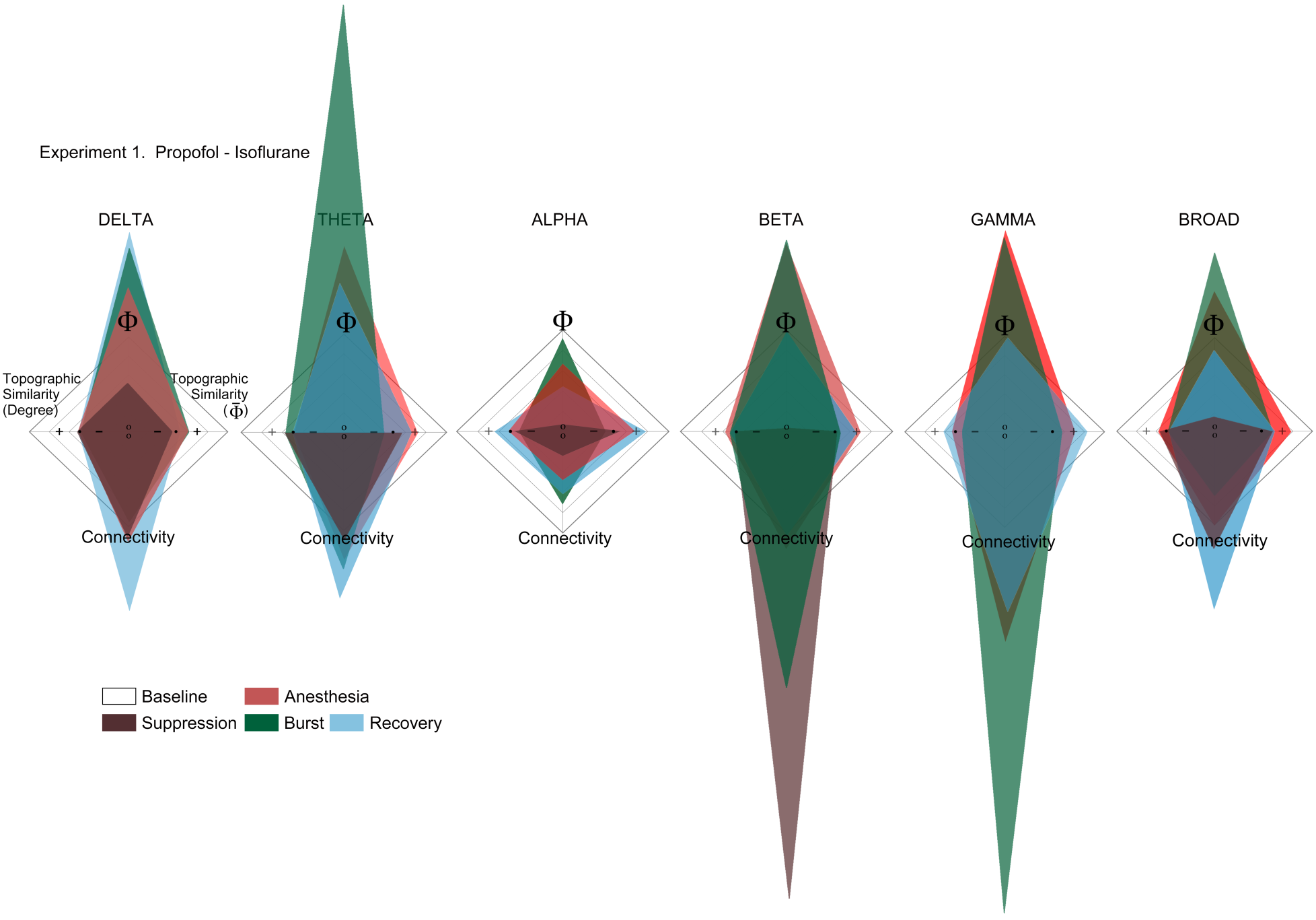


**Supplementary Figure 6.** Multi-dimensional parameter space of propofol-isoflurane experiment for each frequency band.


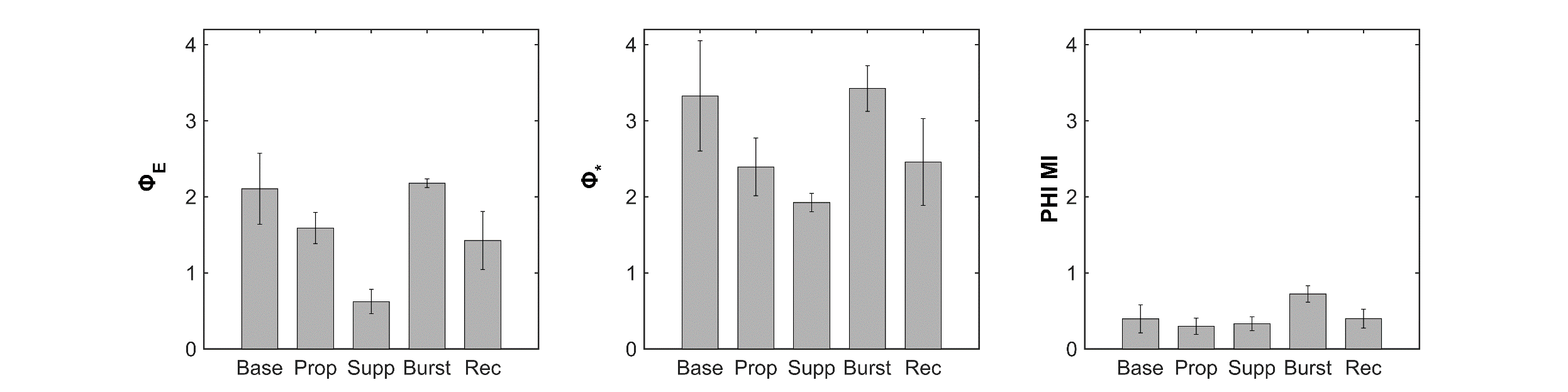


**Supplementary Figure 7.** Comparison between $\tilde{\Phi}_{E}$ and $\Phi^{*}$. The $\tilde{\Phi}_{E}$ (empirical $\Phi$ in Barrett and Seth, 2011 paper) and $\Phi^{*}$ (in Oizumi and Amari et.al., 2016 paper) were calculated by using same partitions of $\tilde{\Phi}_{AR}$ calculation. The both patterns of $\tilde{\Phi}_{E}$ and $\Phi^{*}$ among states are similar with $\tilde{\Phi}_{AR}$. Base=baseline; Prop=propofol; Supp=suppression; Rec=recovery

## Supplementary Tables


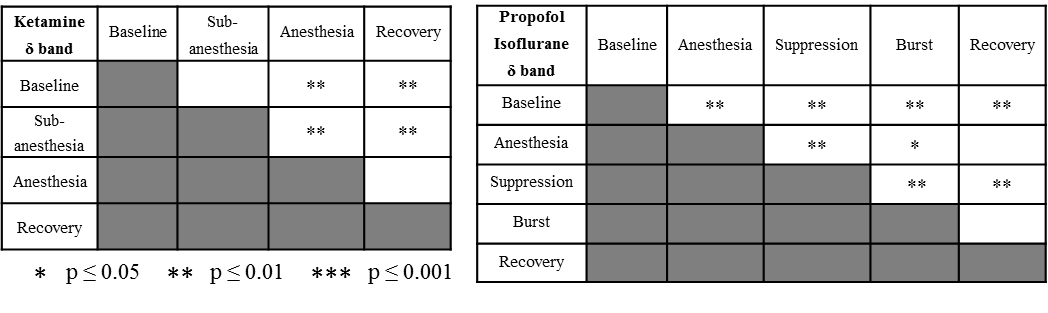


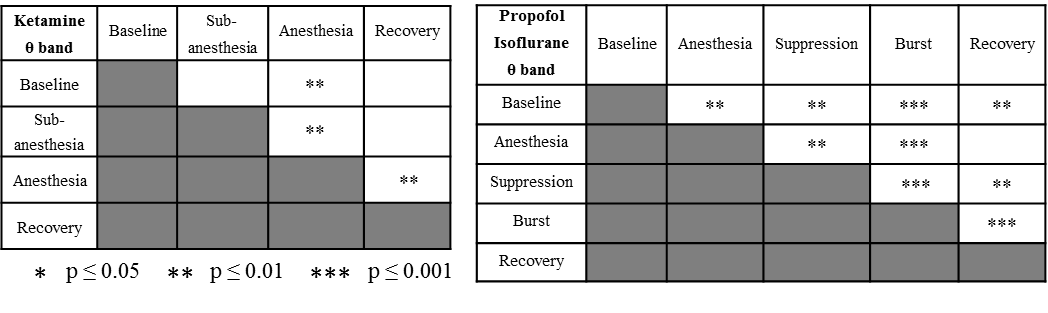


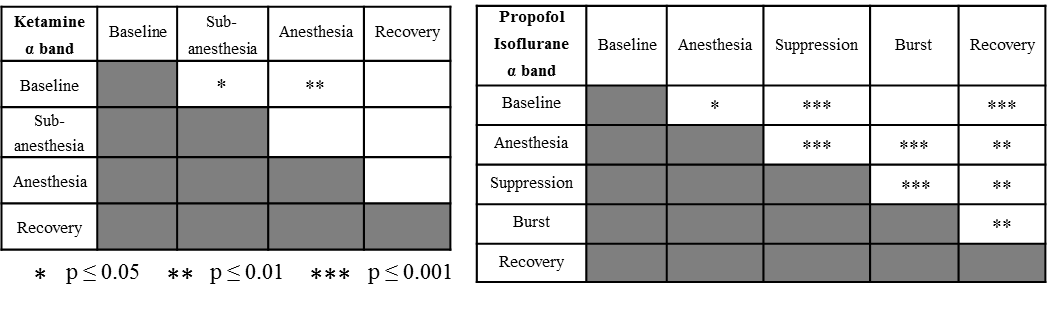


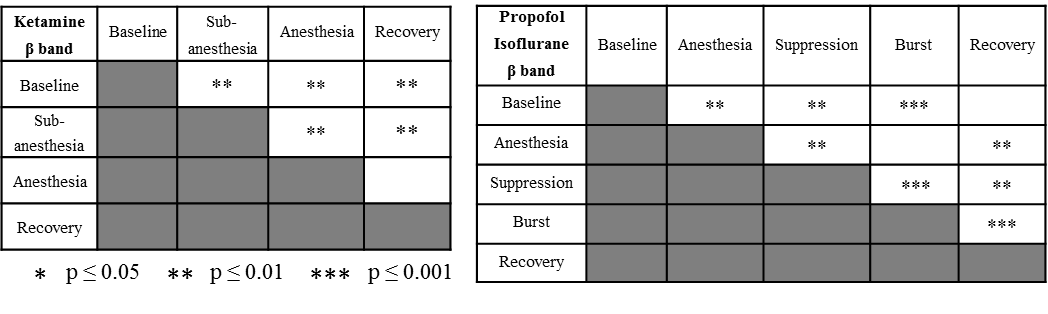


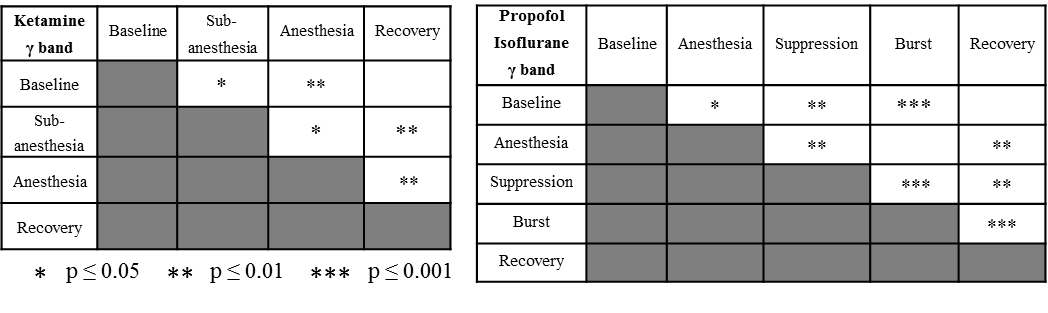


**Supplementary Table 1.** Statistical tests of the change of $\bar{\Phi}$ across the states for each frequency band.


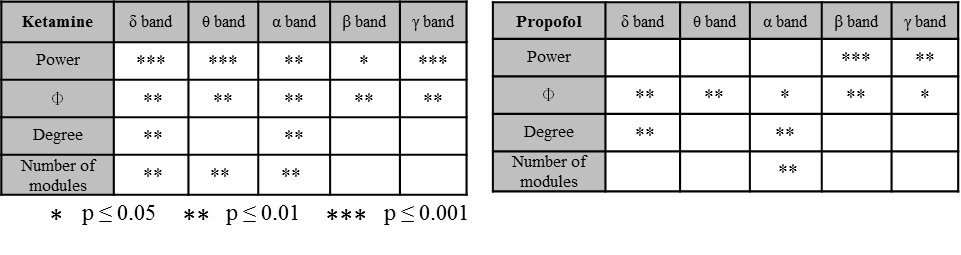


**Supplementary Table 2.** Statistical tests for the differences of power, $\bar{\Phi}$, average node degree, and number of modules between baseline and anesthesia induced by ketamine and propofol.


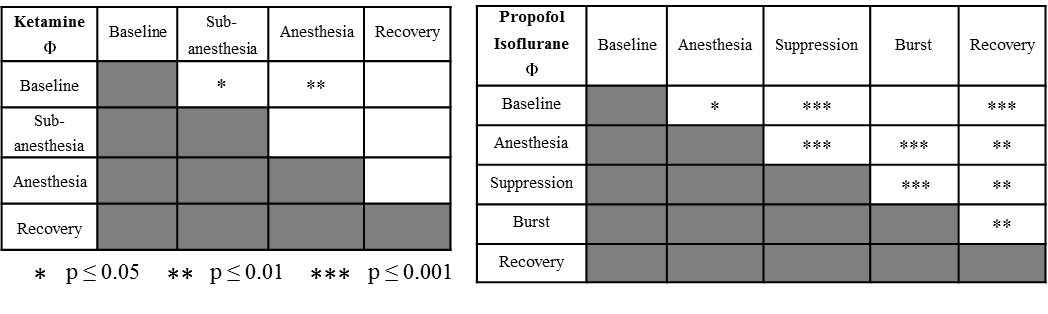


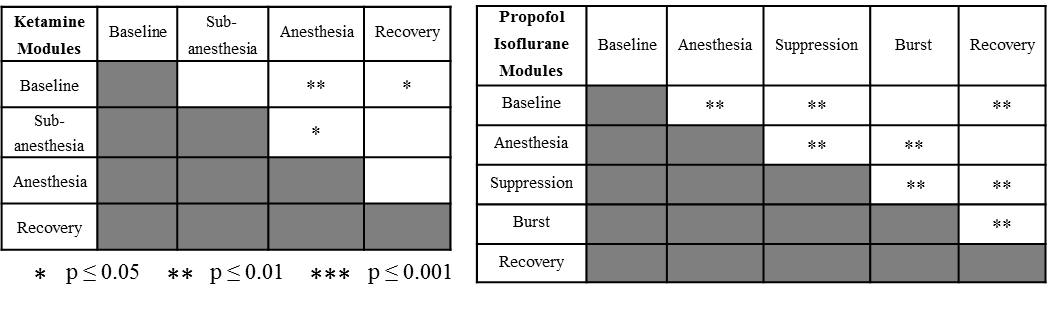


**Supplementary Table 3.** Statistical tests for the difference of $\bar{\Phi}$ and the number of modules across the states for the alpha band.


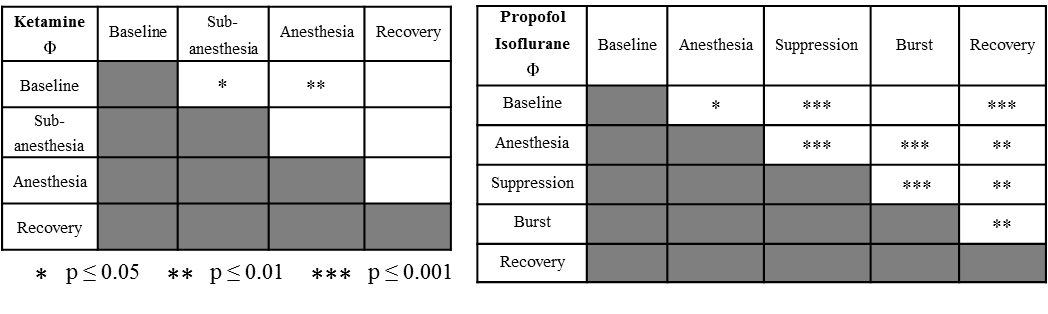


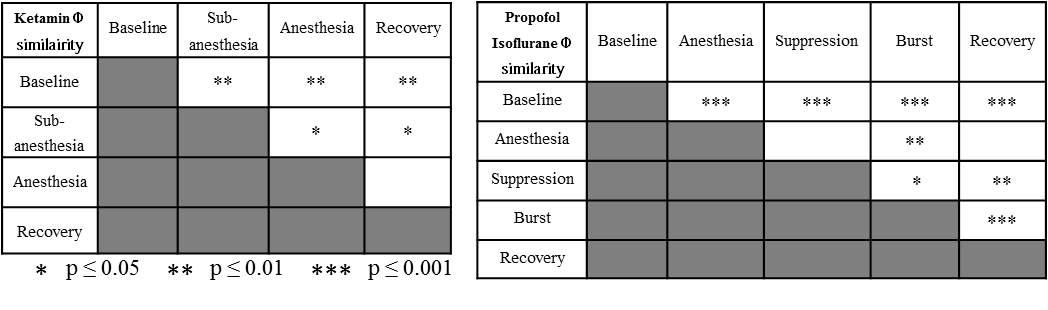


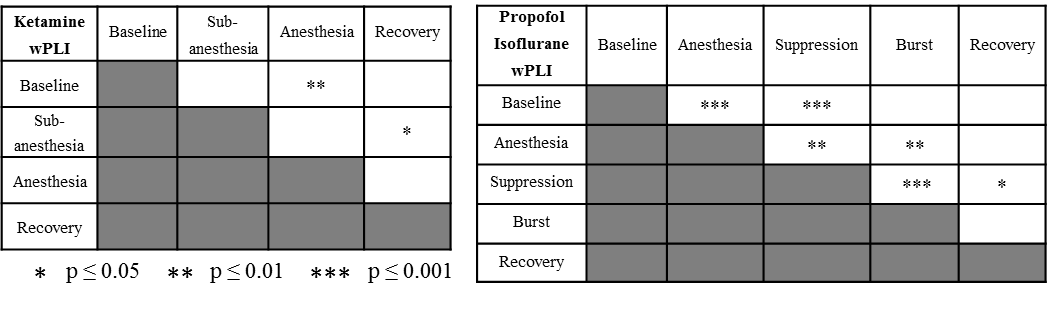


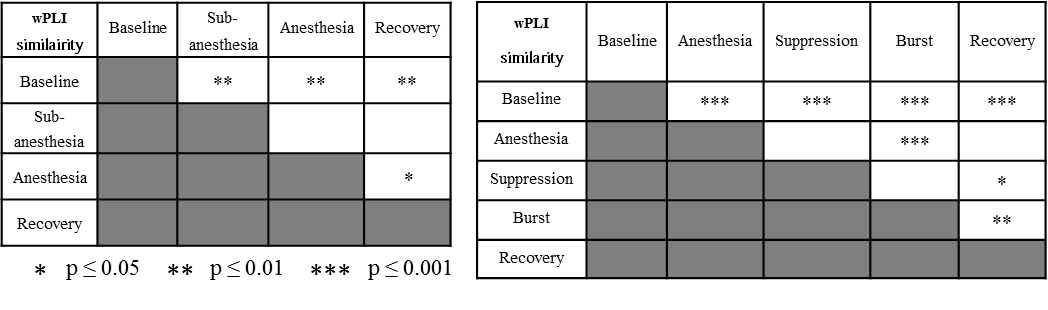


**Supplementary Table 4.** Statistical tests for the difference of a measure across the states in each axis of the multi-dimensional parameter space.
